# SWI/SNF Associates with Nascent Pre-mRNPs and Regulates Alternative Pre-mRNA Processing

**DOI:** 10.1371/journal.pgen.1000470

**Published:** 2009-05-08

**Authors:** Anu Tyagi, Jessica Ryme, David Brodin, Ann Kristin Östlund Farrants, Neus Visa

**Affiliations:** 1 Department of Molecular Biology and Functional Genomics, Stockholm University, Stockholm, Sweden; 2 Department of Cell Biology at the Wenner-Gren Institute, Stockholm University, Stockholm, Sweden; 3 Bioinformatics and Expression Analysis Core Facility, Department of Biosciences and Nutrition, Karolinska Institute, Huddinge, Sweden; European Molecular Biology Laboratory, Germany

## Abstract

The SWI/SNF chromatin remodeling complexes regulate the transcription of many genes by remodeling nucleosomes at promoter regions. In *Drosophila*, SWI/SNF plays an important role in ecdysone-dependent transcription regulation. Studies in human cells suggest that Brahma (Brm), the ATPase subunit of SWI/SNF, regulates alternative pre-mRNA splicing by modulating transcription elongation rates. We describe, here, experiments that study the association of Brm with transcribed genes in *Chironomus tentans* and *Drosophila melanogaster*, the purpose of which was to further elucidate the mechanisms by which Brm regulates pre-mRNA processing. We show that Brm becomes incorporated into nascent Balbiani ring pre-mRNPs co-transcriptionally and that the human Brm and Brg1 proteins are associated with RNPs. We have analyzed the expression profiles of *D. melanogaster* S2 cells in which the levels of individual SWI/SNF subunits have been reduced by RNA interference, and we show that depletion of SWI/SNF core subunits changes the relative abundance of alternative transcripts from a subset of genes. This observation, and the fact that a fraction of Brm is not associated with chromatin but with nascent pre-mRNPs, suggest that SWI/SNF affects pre-mRNA processing by acting at the RNA level. Ontology enrichment tests indicate that the genes that are regulated post-transcriptionally by SWI/SNF are mostly enzymes and transcription factors that regulate postembryonic developmental processes. In summary, the data suggest that SWI/SNF becomes incorporated into nascent pre-mRNPs and acts post-transcriptionally to regulate not only the amount of mRNA synthesized from a given promoter but also the type of alternative transcript produced.

## Author Summary

Genetic programs in multicellular organisms often involve different levels of regulation. The expression of many genes is regulated by factors that remodel the structure of the chromatin at the promoter. SWI/SNF is one such factor, and it is highly conserved in eukaryotes. Studies in human cells suggest that Brahma, the catalytic subunit of SWI/SNF, regulates the processing of precursor mRNAs (pre-mRNAs). We have studied Brahma in two insect model systems to further elucidate the mechanisms by which SWI/SNF regulates gene expression. We show that depletion of SWI/SNF subunits changes the relative abundances of alternative transcripts from a subset of pre-mRNAs that code for proteins that regulate the postembryonic development of the flies. We also show that a fraction of Brahma is not associated with chromatin but with nascent pre-mRNPs—both in insects and mammals—which suggests that SWI/SNF acts at the RNA level to regulate pre-mRNA processing. These findings illustrate the dual role of a chromatin remodelling factor; SWI/SNF acts both at the transcriptional level and post-transcriptionally to regulate not only the amount of mRNA synthesized from a given promoter but also the type of alternative transcript produced.

## Introduction

Messenger RNAs (mRNAs) are synthesized in eukaryotic cells as precursor RNA molecules (pre-mRNAs), which are then assembled into ribonucleoprotein complexes (pre-mRNPs) during transcription. The newly synthesized pre-mRNAs are modified by capping, splicing and 3′-end maturation reactions that involve cleavage and polyadenylation of the 3′-end of the transcript. The existence of alternative splicing and alternative polyadenylation sites in many pre-mRNAs is a major source of protein variability, and the regulation of pre-mRNA processing is a major mode of genetic regulation [1],[2]. Many observations during the last decade have indicated that some of the mechanisms that regulate the processing of the pre-mRNA are related to the transcription process itself, and that chromatin dynamics, transcription and pre-mRNA processing are functionally connected [reviewed in 3].

Transcription can influence the usage of alternative splice sites in the pre-mRNA [4] through several mechanisms. Promoter-specific coregulators can recruit splicing factors to transcribed genes or they can themselves play dual roles in the regulation of transcription initiation and alternative splicing [review by 5]. For example, the hnRNP-like protein CoAA, which coactivates the transcription of multiple genes regulated by steroid hormones [6], is recruited to the promoters of its target genes by the coactivator TRBP/NCoA6 and regulates splice site selection [7]. The CAPER proteins, members of the U2AF65 family, and the polyC-RNA-binding protein 1, PCBP1, are further examples of transcriptional coactivators that influence alternative splicing in a promoter-dependent manner [8],[9]. The RNA polymerase itself can also participate in the recruitment of pre-mRNA processing factors. A reported case is that of SRp20, a member of the SR protein family of splicing regulators. SRp20 is recruited to transcribed genes through interaction with the C-terminal domain (CTD) of the large subunit of RNA polymerase II [10]. It has been proposed that these proteins are recruited to the promoter, travel along the gene with the transcription machinery, and are eventually delivered to the nascent pre-mRNA for splicing regulation [5].

Low transcription elongation rates favor the usage of proximal splice sites by increasing the time during which the proximal sites are exposed to the splicing machinery before more distal sequences are synthesized. This provides a further mechanism by which transcription influences pre-mRNA processing [reviewed in 11].

A recent study has shown that the human Brahma (hBrm) protein, a chromatin remodeling factor, regulates the alternative splicing of several genes in human cells [12]. Overexpression and depletion experiments have shown that hBrm, together with the mRNA-binding protein Sam68, favors the accumulation of RNA pol II at specific gene positions and decreases the elongation rate of the RNA pol II. These effects favor the inclusion of variable exons with weak splice sites [12].

The hBrm protein and its paralog hBrg1 are the ATPase subunits of the SWI/SNF chromatin remodeling complexes in human cells [reviewed in 13]. The SWI/SNF complexes regulate the transcription of many genes in mammalian cells by remodeling nucleosomes at promoter regions [reviewed in 14]. The Brm protein in *Drosophila melanogaster* (dBrm) is associated with transcribed loci in the polytene chromosomes and plays an important role in ecdysone-dependent transcription regulation [15],[16]. Two types of dBrm-containing complexes, BAP and PBAP, are present in *Drosophila*. They share seven core subunits, including dBrm, but differ in the presence of additional signature subunits [17 and references therein].

We have analyzed *in situ* the association of Brm with the actively transcribed Balbiani ring (BR) genes of the dipteran *Chironomus tentans* (*C. tentans*) in order to obtain further insight into the mechanisms by which Brm influences pre-mRNA splicing. The BRs are giant puffs that form by active transcription of the BR genes in the polytene chromosomes of the salivary gland cells [18]. The BR pre-mRNAs synthesized in the BR1 and BR2 puffs have all the features of typical pre-mRNAs. They are capped at the 5′-end [19], spliced, cleaved and polyadenylated at the 3′-end [20], released from the chromosome, and finally exported to the cytoplasm [reviewed in 21]. It is possible to visualize using transmission electron microscopy (TEM) how the BR pre-mRNPs are synthesized along the BR genes [22], and it is possible to study the association of defined proteins with nascent BR pre-mRNP particles *in situ* using specific antibodies.

The Brm protein of *C. tentans* (ctBrm) is associated with the BR puffs and is widely distributed along the active BR genes, as shown by immuno-electron microscopy (immuno-EM) and chromatin immunoprecipitation (ChIP), which suggests that ctBrm has further roles in addition to that of regulating transcription initiation [23]. We have analyzed the location of ctBrm in the actively transcribed BR genes in more detail, and we have shown that a fraction of Brm is associated with the nascent transcripts in both insect and mammalian cells. We have also determined whether Brm plays a role in pre-mRNA processing in insects and we have analyzed the expression profiles of *D. melanogaster* cells in which the levels of dBrm have been reduced by RNA interference (RNAi). We show that depletion of dBrm affects not only the splicing but also the usage of alternative polyadenylation sites.

## Results

### ctBrm Is Associated with the BR Genes

We used three different antibodies against ctBrm to study the association of this protein with the BR genes of *C. tentans*. The first one, Ab1, was raised against the rat Brg1 protein [24]. The second antibody, Ab2, was raised against the C-terminal part of the Ct-BRM protein (Figure S1). The third one, Ab3, was raised against dBrm and has been characterized by Zraly et al. [25]. The specificity of the antibodies was tested by Western blot against nuclear protein extracts prepared from *C. tentans* cultured cells. The three antibodies recognized one major band of approximate molecular mass 200 kDa, the expected molecular mass of ctBrm (Figure S2A). The same band was present in protein extracts prepared from larval salivary glands (Figure S2B). Moreover, the ctBrm protein immunoprecipitated by Ab1 was recognized by Ab2 and by Ab3 (Figure S2C). We concluded that the three antibodies recognized the same protein, ctBrm.

**Figure 1:**
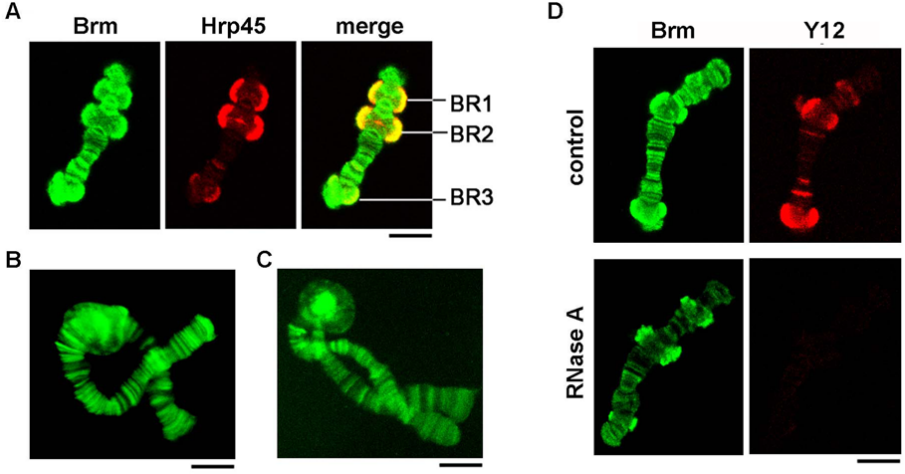
The association of ctBrm with the polytene chromosomes studied by immunofluorescence. (A) The distribution of ctBrm in chromosome IV. Confocal sections of isolated polytene chromosomes stained with the Ab1 antibody and with mAb 2E4 against the mRNA-binding protein Hrp45. CtBrm was associated with multiple loci, including the Balbiani ring puffs BR1, BR2 and BR3 in chromosome IV. (B–C) The association of ctBrm with other chromosomes revealed with Ab2 (B) and Ab3 (C). Both antibodies gave a banded pattern. (D) The association of ctBrm with the chromosomes is partly dependent on interactions with RNA. Polytene chromosomes were isolated and treated with RNase A before fixation and immunostaining (*RNase A*). Control chromosomes were incubated in parallel without RNase A (*control*). The chromosomes were double-stained with Ab1 (*green*) and mAb Y12 against snRNP proteins (*red*). The secondary antibodies were conjugated to FITC and Texas Red, respectively. The scale bars represent 10 µm.

Immunofluorescent staining of isolated polytene chromosomes gave a banded pattern from all three antibodies, and the antibodies stained many chromosomal bands. The actively transcribed BR puffs were among the most intensely stained loci (Figures 1A–C and S3A–D). In some cases, the chromosomes were co-stained with a mAb against Hrp45, an hnRNP protein used as a marker to visualize the BRs in chromosome IV (Figures 1A and S3C–D).

Preparations of isolated polytene chromosomes were digested with RNase A before immunostaining to determine whether the association of Brm with the chromosomes was mediated by RNA (Figure 1D). The chromosomes were co-stained with Y12, a mAb against core snRNP proteins, to monitor the effect of the RNase treatment. Control chromosomes were incubated in parallel in the absence of RNase A. The snRNP staining (*red* in Figure 1D) was abolished by the RNase treatment, as expected. The intensity of the Brm staining (*green* in Figure 1D) was reduced. However, a part of the Brm staining was resistant to the RNase treatment. These results suggest that there are two modes of interaction of Brm with the chromosomes. One mode is independent of the presence of RNA and may be explained by a direct association of Brm with the chromatin. The other mode requires RNA.

**Figure 2:**
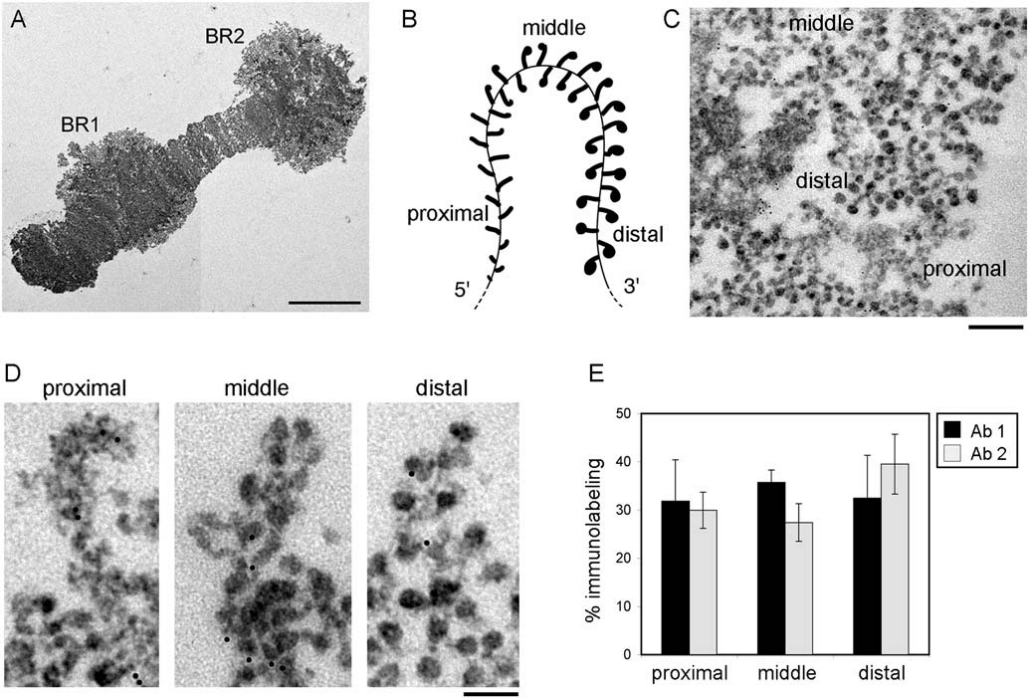
The distribution of ctBrm along the active BR gene analyzed by immuno-EM. (A) Micrograph of a portion of chromosome IV showing the BR1 and BR2 puffs. The scale bar represents 10 µm. (B) Scheme of the active BR transcription unit illustrating the distinct morphology of the growing pre-mRNPs in the proximal, middle, and distal portions of the BR gene. (C) Immuno-electron microscopic analysis of ctBrm distribution in the active BR transcription unit. The image shows a section of a chromosome stained with Ab2 where the proximal, middle and distal portions of the BR gene can be recognized. The bar represents 200 nm. (D) Examples of immuno-EM labeling using Ab2 in the proximal, middle and distal portions of the gene, as indicated. The bar represents 100 nm. (E) Quantitative analysis of the distribution of ctBrm along the BR gene. Comparison of the distribution of gold markers obtained with Ab1 and Ab2. The numbers of immuno-gold markers located in each portion of the gene were counted, and the distribution of labeling was expressed as the percentage of immuno-gold markers in each portion of the gene. Four chromosomes were analyzed for Ab1 and two for Ab2. The error bars are standard deviations. The total number of gold markers analyzed was 5 765 for Ab1 and 1124 for Ab2.

We have previously mapped the association of ctBrm with the BR1 gene by immuno-EM using the anti-Brg1 antibody, Ab1, and we have shown that ctBrm is widely distributed along the entire BR1 gene [23]. We have here confirmed this observation by extending the immuno-EM analysis to BR1 and BR2, where we have used the *C. tentans*-specific antibody Ab2. The results obtained are summarized in Figure 2. The BR1 and BR2 genes are approximately 40 kb long and they are transcribed simultaneously by several RNA polymerases. The different regions of the gene show specific morphological features due to the progressive growth and assembly of the nascent BR pre-mRNPs (Figure 2B). Full-length genes are not available in the sections used for TEM, but partial gene segments are observed (Figure 2C–D). The gene segments can be classified into proximal, middle and distal segments, based on the morphology of the nascent pre-mRNPs. The pre-mRNPs in the proximal region appear as growing fibers with increasing length, whereas the pre-mRNPs in the middle and distal regions appear as stalked granules of increasing diameter.

We isolated chromosome IV from salivary glands and stained the isolated chromosomes with either Ab1 or Ab2. The antibody-binding sites were revealed using a gold-conjugated secondary antibody. Control chromosomes were processed in parallel in order to assess the specificity of the immunolabeling. The chromosomes were embedded in plastic after the immunolabeling and sectioned for TEM analysis. Photographs were taken at random positions, and each gold particle was classified according to its association with a proximal, middle or distal gene segment (Figures 2C and 2D). ctBrm was present on the proximal, middle and distal regions of the BR genes. Similar results were obtained with Ab1 and Ab2 (Figure 2E). These results confirm that ctBrm is widely distributed along the BR genes.

### ctBrm Is Associated with Nascent Pre-mRNPs

We wanted to determine whether the ctBrm protein located at the BR genes was associated with the chromatin or with the nascent BR pre-mRNPs. Detailed analysis of immuno-EM data provides enough resolution to distinguish between labeling of the pre-mRNPs and labeling of the chromatin, as shown by Wetterberg et al. [26].

**Figure 3:**
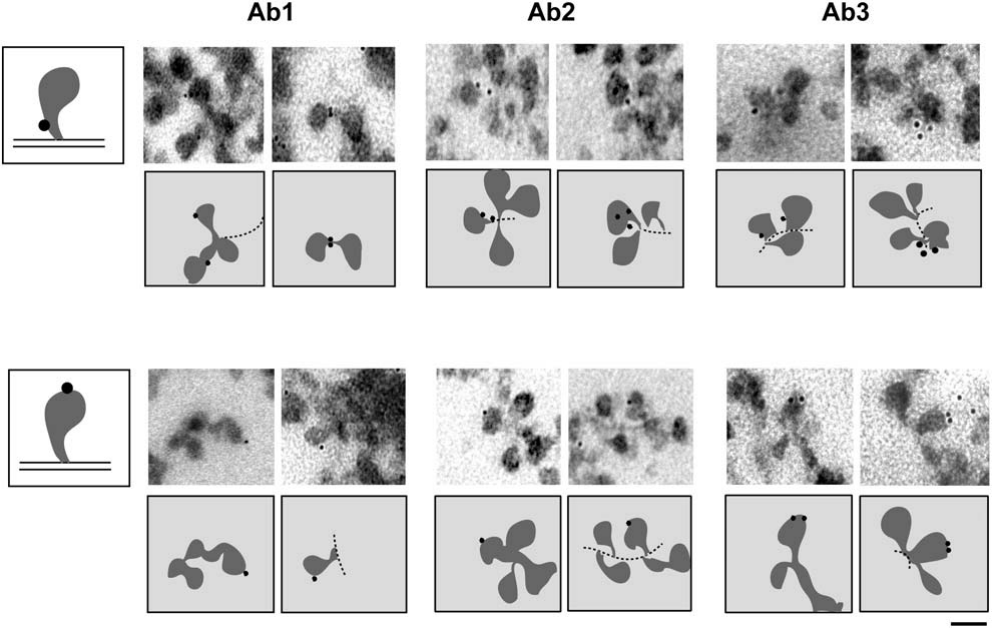
ctBrm is associated with nascent BR pre-mRNP particles. Polytene chromosomes were isolated and immunolabeled with either Ab1, Ab2 or Ab3 against ctBrm, as indicated in the figure. The figure shows four examples of immuno-gold labeling with each of the antibodies. Schematic interpretations of the images are provided under each micrograph. In the top part of the figure, the immuno-gold markers are located close to the chromatin axis (*dotted line*). The examples in the bottom part of the image show nascent BR pre-mRNPs with labeling in the globular domain, far from the chromatin. The single diagrams on the far left at the top and bottom illustrate BR pre-mRNPs with labeling close to the chromatin axis (*top*) and in the RNP globular domain (*bottom*). The scale bar represents 50 nm.

We selected 60 distal BR segments in which the relative positions of the chromatin axis and the nascent pre-mRNPs could be identified, and we determined for each segment whether the gold markers were close to the chromatin (within 50 nm of the axis) or distant from the chromatin (more than 50 nm from the axis). The dimensions of the antibodies mean that this latter group contains only gold markers associated with BR pre-mRNP. In contrast, the markers close to the chromatin may label ctBrm molecules bound to the stalk of the pre-mRNP, bound to the chromatin, or bound to the transcription machinery. The gold markers were distant from the chromatin in 20 out of 60 analyzed cases (33%). A fraction of ctBrm was associated with the nascent pre-mRNPs and this association was confirmed with all three anti-Brm antibodies, as shown in Figure 3. This result agrees with the results of the RNase A digestion experiments shown in Figure 1D. We conclude that a fraction of ctBrm is associated with the nascent BR pre-mRNPs and is not in contact with the chromatin.

**Figure 4:**
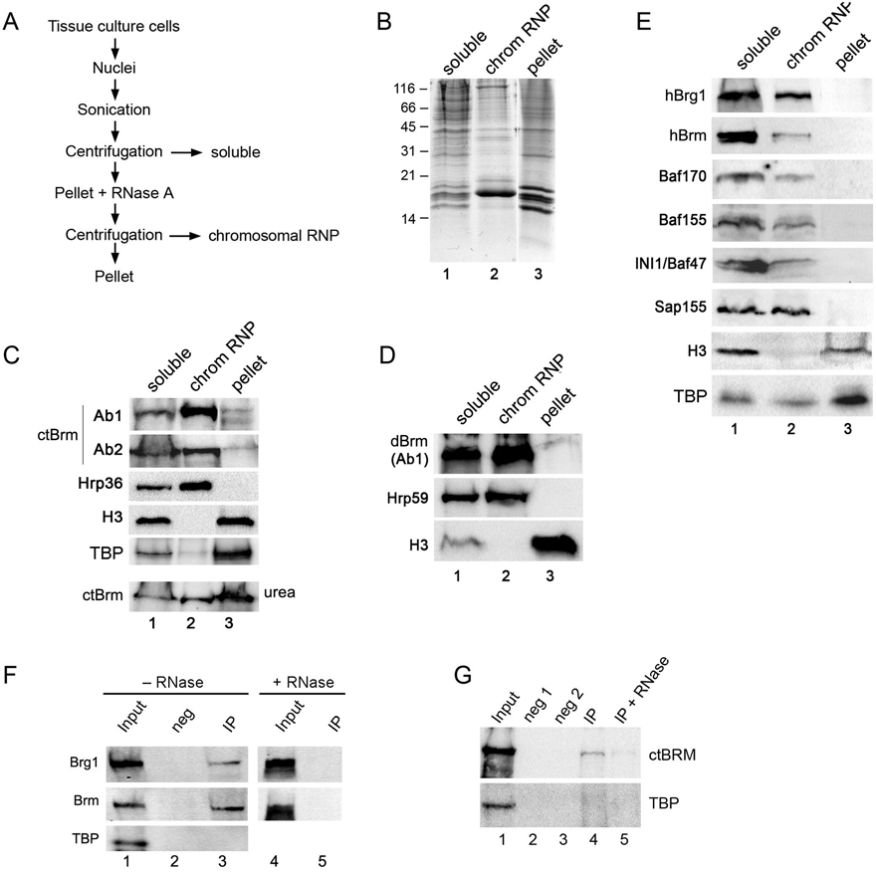
The association of Brm with different nuclear fractions in insect and mammalian cells. (A) Schematic description of the biochemical fractionation scheme. The experimental details are described in the Materials and Methods section. (B) The soluble fraction (*lane 1*), the chromosomal RNP fraction (*lane 2*) and the residual pellet (*lane 3*) prepared from *C. tentans* tissue culture cells were resolved by SDS-PAGE and stained with Coomassie Brilliant Blue to show the protein complexity of each fraction. (C) Nuclear fractions prepared from *C. tentans* tissue culture cells as in (B) were probed with antibodies Ab1 and Ab2 against ctBrm. Antibodies against the hnRNP protein Hrp36, histone H3 and TBP were used in parallel as controls for the composition of the fractions. A significant part of ctBrm was present in the chromosomal RNP fraction (*lane 2*). The presence of ctBRM in the pellet was best revealed in samples dissolved in sample buffer supplemented with 8 M urea (*bottom panel*). (D) Nuclear extracts were prepared from *D. melanogaster* S2 cells following the same fractionation scheme as above. In this case, an antibody against the hnRNP protein Hrp59 was used as a control for the RNP fraction. (E) HeLa cells were fractionated as above and probed with antibodies against different core subunits of SWI/SNF, as indicated. An antibody against Sap155 was used as a control for the RNP fraction. SWI/SNF factors associated with chromosomal RNPs also in HeLa cells. (F) Co-immunoprecipitation of hBrm and hBrg1 with snRNPs. Soluble RNP nuclear extracts were prepared from HeLa cells, treated with or without RNase A as indicated, and used for immunoprecipitation with the Y12 antibody against snRNP proteins (lanes 1–5). The immunoprecipitated proteins were probed by Western blot as indicated to the left. Lanes 2 (*neg*) is a negative control immunoprecipitation processed in parallel. hBrg1 and hBrm are bound to snRNPs (*lane 3*) but the interactions are lost in the RNase-digested extracts (*lane 5*). (G) Soluble RNP nuclear extracts were prepared from *C. tentans* cultured cells and used for immunoprecipitation with the Y12 antibody. An interaction between ctBrm and snRNPs was detected by Western blot with the anti-ctBrm antibody (*lanes 4*). RNase A digestion of the beads before elution drastically reduced the interaction between ctBrm and snRNPs (*lane 5*). *Lanes 2* and *3* are two negative control reactions processed in parallel. The blot was reprobed with the anti-TBP antibody.

We used a cell fractionation assay to confirm the association of ctBrm with RNPs. We isolated nuclei from *C. tentans* tissue culture cells and prepared two types of protein extracts (Figure 4A). One of the extracts contained soluble nuclear proteins (*soluble*) and the other extract contained proteins bound to the chromosomes via RNA (*chromosomal RNP*). These proteins could be released by RNase A digestion. The proteins in each fraction were resolved by SDS/PAGE and analyzed by Western blotting. Coomassie Blue staining showed that each fraction contained a different set of proteins (Figure 4B). Antibodies against Hrp36, an abundant member of the hnRNP A family, histone H3 and the TATA-binding protein (TBP) were used as controls to assess the quality of the fractions. Hrp36 was present in both the soluble and the chromosomal RNP preparations, as expected, whereas histone H3 and TBP were found in the soluble fraction and in the pellet (which contained nuclear components that were not extracted by RNase, such as chromatin and the nuclear envelope). ctBrm was present in the soluble nuclear fraction and in the chromosomal RNP fraction (Figure 4C).

The low abundance of ctBrm in the pellet (*lane 3*, Figure 4C) was unexpected considering that a fraction of ctBrm remained associated with the chromosomes after RNase digestion (Figure 1D). The signal in the pellet was considerably increased by using a sample buffer supplemented with 8 M urea, as shown in the bottom panel of Figure 4C. This observation is consistent with the immunofluorescence experiments and indicates that a fraction of ctBrm is highly insoluble.

We repeated the fractionation experiments using *Drosophila* S2 cells (Figure 4D) and human HeLa cells (Figure 4E). The mRNA-binding proteins Hrp59 and SAP155 were used as RNP controls in *Drosophila* and human extracts, respectively. In S2 cells, dBrm was clearly present in the soluble and chromosomal RNP fractions. In HeLa cells, hBrm and hBrg1 were also present in both soluble and chromosomal fractions. We analyzed the presence of other core SWI/SNF subunits in the fractions from HeLa cells. All the analyzed subunits were present in the soluble fraction, as expected, and all of them were also present in the chromosomal RNP fraction (Figure 4E).

In summary, we conclude that a fraction of the Brm protein is associated with chromosomal RNPs and that the association of Brm with the RNPs is conserved from insects to mammals. Our results also suggest that hBrm and hBrg1 are not bound to nascent RNPs as individual proteins in human cells, but as components of SWI/SNF complexes.

### ctBrm Interacts with snRNP Complexes

The hBrm and hBrg1 proteins interact with snRNPs [12],[27]. We showed that they interact with snRNPs in HeLa cells using immunoprecipitation experiments with the Y12 antibody against core snRNP proteins (Figure 4F). We showed also that ctBrm interacts with snRNP complexes in *C. tentans* (Figure 4G). We prepared a soluble RNP extract, immunoprecipitated snRNPs with Y12, and probed the immunoprecipitated proteins with anti-Brm antibodies. ctBrm was co-immunoprecipiated with snRNPs in the soluble fraction (Figure 4G, lane 4). To determine whether the interaction of ctBrm with snRNPs is a direct protein-protein interaction or whether it requires RNA, the immunoprecipitation was carried out as above and the bound material was treated with RNase A before elution. As shown in Figure 4G, *lane 5*, the signal intensity was significantly reduced in the RNase-treated sample. We assessed the specificity of the interaction by re-probing the blot with an antibody against TBP, a protein that is not expected to interact with snRNPs. We conclude that ctBrm is associated with snRNPs and that the association is RNA-dependent.

### dBrm Affects Pre-mRNA Processing

We next wanted to determine whether Brm affects pre-mRNA processing in *Drosophila*. Moshkin and coworkers have determined the expression profiles of *Drosophila* S2 cells after depletion of individual SWI/SNF subunits by RNAi and microarray hybridization using the Affymetrix *Drosophila* Genome 2 arrays. Three independent RNAi experiments followed by RNA extraction and microarray hybridization were carried out for dBrm, and six independent experiments were carried out for mock-treated cells [28]. The data from these experiments is available at Array Express, E-TABM-169 (http://www.ebi.ac.uk/microarray-as/aew/). We investigated the effects of dBrm depletion on the relative abundances of alternatively spliced transcripts by mining the E-TABM-169 data and selecting those genes that were represented by more than one probe set in the *Drosophila* Genome 2 arrays (974 genes). In many cases, the multiple probe sets targeted different parts of the same transcript, pseudogenes or alternative transcripts derived from alternative promoters of the same gene. We found evidence that SWI/SNF regulates the activity of many gene promoters, as expected (not shown). We could also identify genes for which the multiple probe sets targeted transcripts that had originated by alternative splicing or alternative polyadenylation of a single pre-mRNA. We selected those genes that displayed changed expression levels specific for at least one transcript with p<0.02. Fifteen of these genes showed transcript-specific expression changes in the dBrm-depleted cells (Table 1). We then used the annotations available at FlyBase (http://flybase.bio.indiana.edu/) to analyze the qualitative differences between the transcripts affected. The transcripts affected show differences in their patterns of alternative, including the use of alternative 3′ slice sites, exon skipping and intron retention (Figure S4). However, the most striking observation was that the processing of the affected transcripts also involved the alternative use of polyadenlation signals, which suggests that dBrm influences not only the splicing but also the formation of the 3′-end of the transcripts.

**Table 1:** Transcripts affected by dBrm depletion in S2 cells(1).

Gene	Transcript Detected(2)	Ratio (3)	p-value(3)	Effect
CG11154 (ATPsyn-beta)	CG11154-RA	0.6	0.004	A down
	CG11154-RA+RB	0.8	0.089	
CG11491 (br)	CG11491-RA	0.7	0.218	
	CG11491-RF	0.1	0.001	RF down
	CG11491-RD	1.6	0.182	
CG12052 (lola)	CG12052-RL	1.5	0.004	RL up
	CG12052-RJ	0.8	0.254	
	CG12052-RF	2.2	0.133	
	CG12052-RS+RZ	1.5	0.050	
	CG12052-RJ	0.9	0.463	
	CG12052-RA	1.5	0.001	RA up
CG18251 (Msp-300)	CG18251-RB	7.3	0.001	RB up
	CG18251-RE	1.0	0.480	
CG19086	CG1906-RB	2.1	0.120	
	CG1906- all	2.2	0.152	
	CG1906-RE	2.8	0.011	RE up
CG2249	CG2249-RA+RB	0.8	0.015	RB down
	CG2249-RA	1.0	0.488	
CG32491 (mod(mdg4))	CG32491-RK	1.1	0.373	
	CG32491-RT	0.9	0.377	
	CG32491-RC	1.3	0.200	
	CG32491-RA	0.5	0.009	RA down
	CG32491-RA+RF	0.4	0.009	RA/RF down
	CG32491-RN	0.9	0.250	
	CG32491-RY	1.3	0.190	
	CG32491-RF	0.6	0.012	RF down
	CG32491-RD	0.5	0.013	RD down
CG3665 (Fas2)	CG3665-RA+RB+RC	2.6	0.166	
	CG3665-RC	2.9	0.002	RC up
	CG3665-RA	0.7	0.204	
CG4859 (Mmp1)	CG4859-RC	4.0	0.006	RC up
	CG4859-RC+RD	3.5	0.012	
	CG4859-RD	1.7	0.117	
CG5174	CG5174- all	1.4	0.174	
	CG5174-RK	2.1	0.015	RK up
	CG5174-RA+RB+RH+RI	1.2	0.333	
CG6899 (Ptp4E)	CG6899-RB	1.2	0.317	
	CG6899-RA+RB	1.6	0.109	
	CG6899-RA	1.9	0.016	RA up
CG8092(4)	CG8092-RA+RB	0.7	0.081	
	CG8092-RB	0.5	0.008	RB down
	CG8092-RA	1.5	0.200	
CG8421 (Asph) (4)	CG8421-RD+RE	1.0	0.455	
	CG8421-RA+RB+RD	2.8	0.003	RA/RB up
CG8676 (Hr39)	CG8676-all	2.3	0.001	RA up
	CG8676-RA+RB+RD	2.2	0.0005	RA up
	CG8676-RC	1.4	0.212	
CG9380(4)	CG9380-RB	17.2	0.0001	RB up
	CG9380-RC	6.4	0.005	RC up

**Figure 5:**
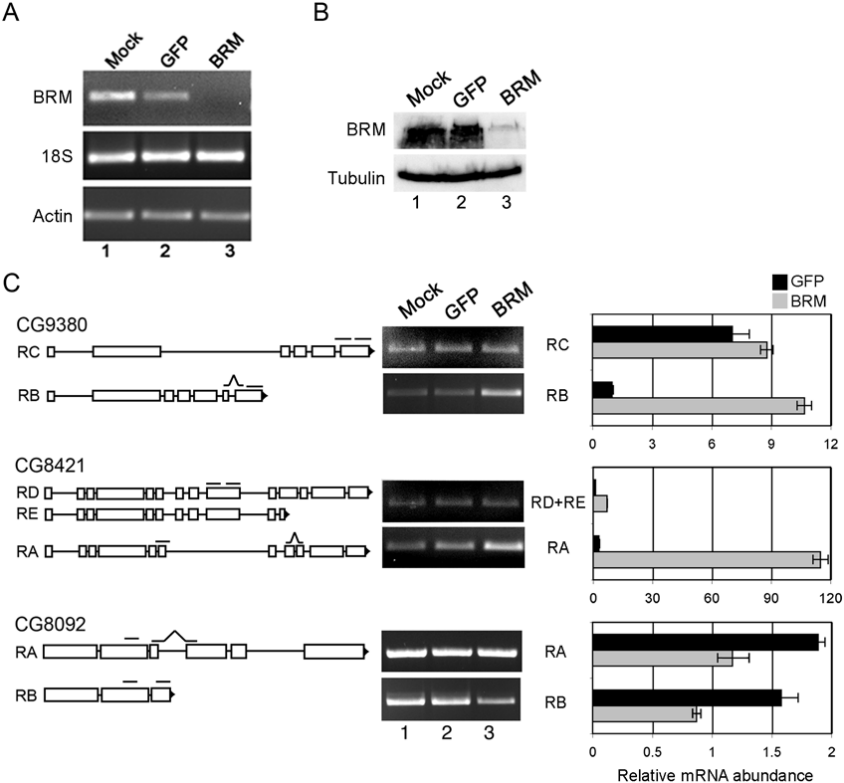
Depletion of dBrm changes the relative abundances of alternatively processed transcripts in S2 cells. S2 cells were treated with dsRNA for dBrm for 4 days. Control experiments were carried out with dsRNA for GFP. Mock-treated cells without dsRNA were also processed in parallel. (A) The efficiency of the depletion was analyzed at the RNA level by RT-PCR. The levels of 18S rRNA and actin 5C mRNA were analyzed in parallel to assess the specificity of the dsRNA treatments. (B) The dsRNA treatment was also effective at the protein level as shown by Western blotting. (C) The effect of dBrm depletion on the processing of pre-mRNA from three selected genes – CG9380, CG8421 and CG8092 – was validated by RT-PCR. Semi-quantitative RT-PCR reactions were analyzed in agarose gels stained with ethidium bromide. The results were also validated by qPCR and expressed as relative abundance related to actin 5C. The exon-intron organization of each transcript is shown to the left. Total RNA was purified from mock-treated cells (*lane 1*) and from cells treated with dsRNA for either GFP (*lane 2*) or dBrm (*lane 3*). The RNA preparations were analyzed by RT-PCR using primer-pairs designed to amplify specific transcripts, as indicated in the figure. The positions of the PCR primer pairs are indicated as short bars on top of each transcript. For each gene, dBrm depletion changed the levels of specific transcripts in agreement with the microarray data. In CG9380 and CG8421, the RB and RA transcripts, respectively, were upregulated in dBrm-depleted cells. In CG8092, the RB transcript was downregulated by dBrm depletion.

(1) Data from Array Express E-TABM-169. See the text for details.

(2) For genes with many alternative transcripts, only a selection of all the available transcripts is presented in the table.

(3) Mean ratios and p-values corresponding to comparisons between three independent dBrm-depletion experiments and six independent mock RNAi experiments.

(4) Genes validated by RT-PCT. See Figure 5.

We validated the microarray results by silencing the expression of dBrm in S2 cells and analysing the expression of four selected genes in which the absence of dBrm affected pre-mRNA processing in different ways, according to the microarray experiments. Mock-treated cells and control cells treated with dsRNA for GFP were analyzed in parallel to assess the specificity of the depletion effects. The levels of dBrm RNA and protein were significantly reduced after 4 days of treatment with dsRNA, as shown by RT-PCR and Western blot, respectively (Figure 5A–B). We designed PCR primers for each of the selected genes in order to amplify specific transcripts and we analyzed the effects of dBrm depletion by RT-PCR. The results of the RT-PCR analyses agreed with the microarray data (Table 1, Figures 5C and S5). In summary, depletion of dBrm affects the relative abundances of alternatively spliced and/or alternatively polyadenylated transcripts.

**Figure 6:**
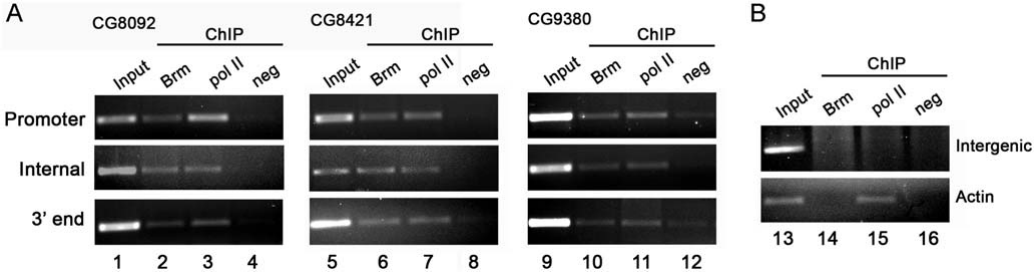
The association of dBrm with its target genes studied by ChIP. (A) Chromatin was extracted from S2 cells after fixation with formaldehyde, and immunoprecipitation reactions were carried out with Ab1 to precipitate chromatin fragments bound to dBrm (*lanes 2*, *6*, *10* and *14*). A positive control immunoprecipitation was carried out using an antibody against the C-terminal domain of the largest subunit of RNA pol-II (*lanes 3*, *7*, *11* and *15*). A negative control immunoprecipitation was carried out in parallel with an unrelated rabbit antibody against mouse immunoglobulins (*lanes 4*, *8*, *12 and 16*). For each gene analyzed, PCR reactions were carried out with primers specific for the proximal promoter, the internal region involved in the alternative processing and the 3′-end of the gene, as indicated in the figure. (B) The actin 5C gene and an intergenic region were analyzed in parallel to assess the specificity of the ChIP (*lanes 13–16*).

Are the effects of SWI/SNF depletion on pre-mRNA processing direct or indirect? Pre-mRNA splicing often occurs co-transcriptionally [20],[29]. We thus asked whether dBrm was associated with the gene regions involved in the alternative processing events that were affected by dBrm depletion, and we carried out chromatin immunoprecipitation (ChIP) experiments to analyze the association of dBrm with the three genes shown in Figure 5. ChIP can detect proteins that are bound to the DNA as well as proteins associated with the nascent pre-mRNA [see for example 30]. For each gene analyzed, we used primer-pairs to detect the proximal promoter, the internal region affected by the alternative processing and the 3′-end of the gene (Figure S6). dBrm associated with all regions of the three genes (lanes 2, 6 and 10 in Figure 6). We used an antibody against the C-terminus of the largest subunit of RNA pol-II as a positive control for the ChIP reactions (lanes 3, 7 and 11 in Figure 6). An unrelated anti-rabbit antibody was used as a negative control (lanes 4, 8 and 12 in Figure 6). Additional controls were carried out by analyzing the association of dBrm with the actin gene, a housekeeping gene whose expression is not regulated by SWI/SNF. The RNA pol-II (lane 15) associated with the actin gene while the dBrm did not (lane 14 in Figure 6). An intergenic region located far from any annotated genes was devoid of both dBrm and RNA pol-II.

**Figure 7:**
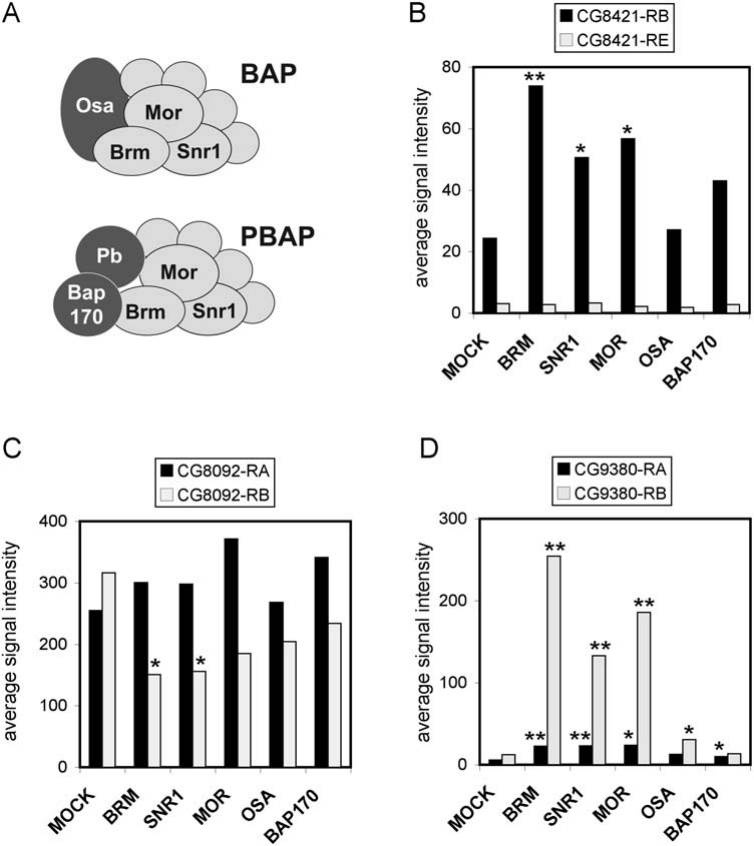
Depletion of other SWI/SNF subunits changes the relative abundances of alternatively processed transcripts in S2 cells. (A) Schematic representation of the composition of the BAP and PBAP complexes. The light grey subunits constitute the SWI/SNF core whereas the dark grey ones represent signature subunits. (B–D) The histograms show the abundances (expressed as average signal intensities) for alternatively processed transcripts from three selected genes, as indicated, in mock-treated cells and in cells treated with dsRNA for Brm, Snr1, Mor, Osa or Bap170. Data from ArrayExpress E-TABM-169 [28]. * indicates p<0.05. ** indicates p<0.005.

We next asked whether dBrm alone or the entire BAP/PBAP complex is responsible for the effects detected at the level of pre-mRNA processing. We mined the data from the E-TABM-169 microarray experiment and asked whether depletion of other SWI/SNF subunits had any effects on the processing of the pre-mRNAs derived from the CG8092, CG8421 and CG9380 genes. Depletion of either Mor or Snr1, two SWI/SNF core subunits, induced changes very similar to those induced by dBrm, whereas depletion of the signature subunits Osa, Bap170 or PB gave milder and in many cases non-significant effects (Figure 7). The effect of Snr1 depletion on the abundances of the CG8421 and CG9380 transcripts was validated by RNAi and RT-qPCR (Figure S7). These results suggest that dBrm does not regulate pre-mRNA processing alone: it is part of the core SWI/SNF complex.

### The Ontology of the Genes Regulated Post-Transcriptionally by SWI/SNF

The 15 genes identified above were tested for enrichment of gene ontology (GO) terms for biological processes and molecular functions [31]. The expected number of genes associated with a given term by random was compared with the observed number of SWI/SNF-regulated genes that were associated with that particular term using the Fisher's exact test. Several GO terms for biological processes were very significantly enriched, including positive regulation of developmental process (GO:0051094, p = 0.00003), programmed cell death (GO:0008219, p = 0.0005) and instar larval or pupal morphogenesis (GO:0048707, p = 0.0008).

The test for enrichment of molecular functions also revealed significant associations. Seven of the genes are predicted to code for proteins with catalytic activity. Three of them have phosphatase activity (GO:0016791, p = 0.0009) and three have RNA polymerase II transcription factor activity (GO:0003702, p = 0.002). And nine out of the fifteen gene products were found to have metal ion binding activity (GO:0046872, p = 0.00002). In agreement with this finding, a search for conserved protein domains revealed that five of the genes regulated by SWI/SNF post-transcriptionally, including the known transcription factors broad/CG11491, lola/CG12052, mod(mdg4)/CG32491 and hr39/CG8676, code for proteins that contain zinc finger domains.

In summary, the tests for ontology enrichments indicate that the genes that are regulated post-transcriptionally by SWI/SNF are primarily enzymes and transcription factors that function in the regulation of postembryonic developmental processes. It is worth mentioning that one of them, broad/CG11491, is a key regulator of metamorphosis [32].

## Discussion

### Brm Binds to the Nascent Pre-mRNP during Transcription

**Figure 8:**
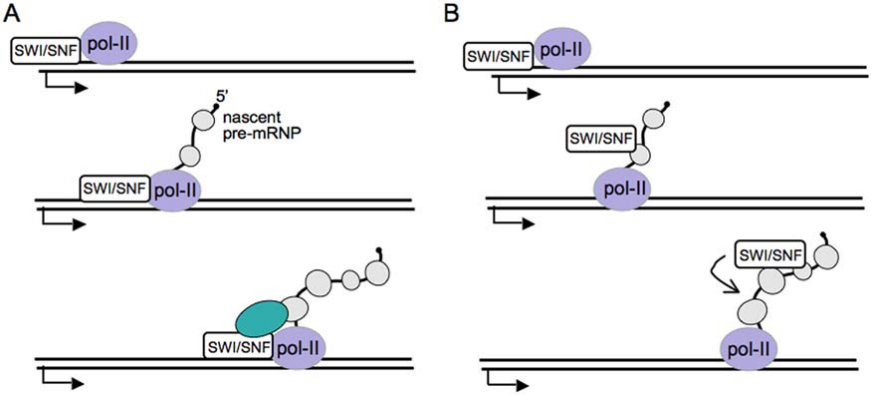
Two models for the post-transcriptional role of SWI/SNF in pre-mRNA processing. (A) SWI/SNF interacts with components of the nascent pre-mRNP complex and with the transcription machinery. These interactions cause a delay in the transcription elongation rate, which in turn affects splice site selection. Based on data from Batsché et al. [12]. (B) SWI/SNF becomes incorporated into the nascent pre-mRNP complex and modulates the interactions between the pre-mRNP and the processing machineries.

The hBrm and hBrg1 proteins are the catalytic subunits of the SWI/SNF chromatin remodeling complexes and much of what is known about their function comes from studies of transcriptional regulation [33],[34]. SWI/SNF participates in regulatory networks that can result in either the activation or the repression of a gene, depending on the genomic context and the activities of additional co-regulators [35]. One of the functions of SWI/SNF is to remodel the structure of nucleosomes at promoter regions in an ATP-dependent manner [36],[37]. In some genes, SWI/SNF is associated also with downstream regions of the genes and influences transcription elongation [38]. Recent studies have shown that hBrm and hBrg1 regulate the alternative splicing of several pre-mRNAs in human cells [12],[39]. The current proposed model (Figure 8A) suggests that hBrm acts together with mRNA-binding proteins such as Sam68 or p54^nrb^ to decrease the elongation rate of RNA pol II and to induce the accumulation of RNA pol II at specific positions in the gene. This facilitates the assembly of the splicing machinery at weak splice sites, which favors the inclusion of proximal exons [12],[39]. Inactivation of the ATPase activity of hBrm does not affect its ability to regulate alternative splicing [12], which indicates that the mechanism by which hBrm regulates pre-mRNA processing is independent of its nucleosome remodeling activity.

We have now studied Brm in two insect model systems, *D. melanogaster* and *C. tentans*, and our results show that Brm becomes incorporated into nascent pre-mRNPs during transcription. This conclusion is based on several observations. Firstly, immunofluorescence experiments combined with RNase A digestion show that the association of ctBrm with the polytene chromosomes of *C. tentans* is partially mediated by RNA. Secondly, immuno-EM reveals that a fraction of ctBrm is associated with the BR pre-mRNPs, not with the chromatin. Thirdly, biochemical fractionation experiments show that Brm is present in the chromosomal RNP fraction in *C. tentans*, *D. melanogaster* and *H. sapiens*. The fact that Brm interacts directly with the nascent pre-mRNP suggests that Brm regulates gene expression post-transcriptionally.

An interesting question is whether the post-transcriptional role of Brm is mediated by the Brm protein alone or in complex with other SWI/SNF subunits. Fractionation of nuclear extracts from HeLa cells showed that several SWI/SNF core subunits, Brm, Brg1, Baf155, Baf170 and INI1/SNF5, are associated with nuclear RNPs. This observation suggests that, at least in human cells, the hBrm and hBrg1 proteins that are present in the RNP-associated fraction are part of a SWI/SNF complex. This seems to be the case also in insect cells, as judged by the effects of depletion of individual SWI/SNF subunits on pre-mRNA processing (see below).

### SWI/SNF Regulates the Abundances of Alternatively Processed Transcripts

We have analyzed the expression profiles of S2 cells in which either Brm or other subunits of SWI/SNF have been silenced by RNAi, and we have identified 15 genes that show changes in the relative abundance of alternatively processed transcripts. The number of genes that are affected by dBrm depletion is likely to be underestimated because our study is based on the use of gene expression arrays that do not fully cover all the transcriptome. Our results clearly show that Brm influences the levels of alternatively processed mRNAs in *Drosophila* cells. Interestingly, the analysis of the expression profiles of S2 cells depleted of other SWI/SNF core subunits revealed effects similar to those induced by Brm depletion. This finding suggests that the role of Brm in pre-mRNA processing is mediated by a core SWI/SNF complex.

What is the functional significance of the alternative processing events regulated by SWI/SNF? For some of the identified genes, the alternative transcripts code for different protein isoforms. For instance, the CG8092 gene encodes two different proteins. The longer isoform, CG8092-PA, contains an AT-hook motif and a zinc finger domain whereas the shorter isoform, CG8092-PB, lacks the zinc finger. Considering that CG8092 is an essential gene in *D. melanogaster* (http://flybase.org/reports/FBal0211894.html) and that the CG8092 proteins resemble transcription factors, changes in the relative abundance of the CG8092 isoforms are likely to be biologically significant.

In other cases, the differences among the alternative transcripts regulated by SWI/SNF lie outside the ORF, in the 3′ UTRs of the mRNAs. The proteins encoded are thus identical, but the stability of the transcripts may differ. A search in miRBase (http://microrna.sanger.ac.uk), a database for microRNA (miRNA) data, revealed that several of the genes identified in our study are predicted targets for miRNA regulation and that in most cases the miRNAs are specific for each alternative transcript (data not shown). One example of this is broad/CG11491, a gene with seven alternative mRNAs with five different 3′ UTRs. Interestingly, all five 3′ UTRs contain predicted miRNA targets [40]. These observations lead us to speculate that in some cases the regulation of alternative processing mediated by SWI/SNF acts in concert with the miRNA pathway to fine-tune the abundances of key proteins with catalytic and/or regulatory activities.

### Transcription versus Post-Transcriptional Regulation

Depletion of SWI/SNF does not change the levels of all the mRNAs that have originated from a pre-mRNA in the 15 genes identified, but only a few. Indeed, in most cases only one mRNA is affected, which indicates that the step that is affected is not only the synthesis of the pre-mRNA but its processing into alternative mRNAs.

SWI/SNF plays an important role in the transcription of many genes, and it may be that the alterations of pre-mRNA processing that we have observed are a consequence of alterations in the synthesis of specific pre-mRNA processing factors. However, we have shown by ChIP that dBrm is physically associated with both the proximal promoter and downstream sequences of the genes affected. We have also shown by immuno-EM of the BR genes of *C. tentans* that ctBrm is associated with nascent pre-mRNPs. These observations strongly suggest that Brm acts directly at the mRNA level.

Our results indicate that dBrm affects both alternative splicing and alternative polyadenylation sites. Many of the alternative processing events regulated by SWI/SNF involve the use of mutually exclusive splicing and polyadenylation sites. The use of the proximal site is favored by dBrm in some of the genes, as would be expected if dBrm acts by reducing the elongation rate of the Pol-II at certain positions, as has been proposed for the regulation of the CD44 pre-mRNA in human cells [12]. However, depletion of dBrm has the opposite effect in other cases (such as CG18251, CG9380, CG3665), which is difficult to reconcile with a model of kinetic regulation based on modulation of the Pol-II elongation rate. This observation, and the finding that a significant fraction of Brm is associated with nascent pre-mRNPs, lead us to propose that dBrm regulates pre-mRNA processing in a more direct manner. Several mechanisms can be envisioned by which Brm, being part of the pre-mRNP complex, could influence the usage of alternative splicing or polyadenylation sites (Figure 8B). In one possible scenario, SWI/SNF could work as an RNP remodeling factor to modulate interactions between specific processing factors and their target RNA sequences. However, this possibility is unlikely because experiments in human cells suggest that the role of hBrm in pre-mRNA processing does not require a functional ATPase domain [12]. Alternatively, SWI/SNF could influence the structure of the pre-mRNP. For instance, SWI/SNF could recruit pre-mRNA processing factors to the nascent transcript, or prevent the interactions of processing factors with their target RNA sequences. The interaction of Brm with each pre-mRNP is likely to depend on the sequence of the transcript and/or the specific combination of proteins that forms the pre-mRNP. As a consequence, the action of Brm and the specific outcome of the processing reactions will depend in each case on the specific features of the pre-mRNP. The same type of context-dependent mechanism has been proposed to explain the complex function of SWI/SNF in transcription regulation [35 and references therein]. SWI/SNF acts as a co-activator in the transcription of certain genes, but represses the transcription of certain other genes [for example 41], and these two opposite effects are mediated by interactions with different types of co-regulators. In a similar manner, SWI/SNF can either repress or activate the choice of splice sites and/or polyadenylation sites in a gene-specific manner.

The role of SWI/SNF in pre-mRNA processing affects a specific subset of pre-mRNAs [12 and our present results]. Ontology analysis revealed that many of the transcripts regulated post-transcriptionally by SWI/SNF in *D. melanogaster* code for proteins that are implicated in postembryonic developmental processes. One of them, broad, is a transcription factor that plays a central role in the cross-talk between ecdysone and juvenile hormone, the two hormones that coordinate insect growth and development [reviewed in 42] Dubrovsky 2005. This is particularly interesting, since hBrm and hBrg1 have also been identified as key regulators of growth control and differentiation in mammals [43],[44]. hBrm and hBrg1 are differentially expressed during development, and their expression is altered in cancer cells, which leads to deregulation of genetic programs [reviewed in 45]. In summary, SWI/SNF appears to act both transcriptionally and post-transcriptionally to fine-tune the expression of genes with key regulatory functions in development. In this way, SWI/SNF can regulate gene expression at two levels by determining not only the amount of mRNA synthesized from a given promoter but also the type of alternative transcript produced. Acting at the pre-mRNA processing level, SWI/SNF can rapidly modulate the abundance and activity of the resulting protein products by acting on genes that are already active.

## Materials and Methods

### Animals and Cell Culture

*Chironomus tentans* were cultured as described by Meyer et al. [46]. The salivary glands used for study were isolated from 4^th^ instar larvae. *C. tentans* tissue culture cells were grown in ZO medium at 24°C as described by Wyss et al. [47]. *D. melanogaster* S2 cells were cultured at 28°C in Schneider's medium (Invitrogen).

### Isolation and Expression of a cDNA That Encodes ctBrm

Degenerate primers for nested PCR were designed based on conserved residues in the C-terminal part of *D. melanogaster* Brm (CG5942) and *Anopheles gambiae* Brm (AGAP010462). The sequences of the primers are provided in the Supplementary Materials and Methods. A PCR product of about 750 bp was amplified from a total cDNA preparation made from *C. tentans* tissue culture cells. The sequence of the PCR product encoded a partial protein corresponding to amino acids 1252–1455 in dBrm. The PCR product was cloned into pET21b (Novagen), expressed in BL21 *E. coli* cells (Novagen) and used to immunize rabbits.

### Antibodies

The antibody against the C-terminal part of ctBrm was raised in rabbit following standard procedures (AgriSera, Sweden). The anti-rat Brg1 antibody was raised and characterized by Östlund Farrants et al. [24]. The anti-dBrm antibody was raised and characterized by Zraly et al. [25]. The antibodies against hBrm, Baf47/INI1, Baf180, Baf155, Baf170 and Sap155 were characterized by Sif et al. [48], de la Serna et al. [49], Nicolas et al. [50], Shanahan et al. [51] and Will et al. [52], respectively. The anti-Baf170 was from Santa-Cruz (sc-10757). The mAbs 10:3G1 and 2E4 against Hrp36 and Hrp45, respectively, have previously been characterized [53],[54]. The Y12 antibody against the Sm epitope of snRNP core proteins was characterized by Lerner et al. [55]. The rabbit anti-Hrp59 was the Y38 antibody raised by Falk et al. [56]. The anti-Pol-II antibody was purchased from Abcam (Ab5408). The anti-TBP was from Santa-Cruz Biotechnology (sc-204). FITC-conjugated, Texas Red-conjugated and gold-conjugated secondary antibodies were from Jackson ImmunoResearch Laboratories. The secondary antibodies conjugated to alkaline phosphatase and horseradish peroxidase were from DakoCytomation.

### Immunofluorescence

Salivary glands were pre-fixed with 2% formaldehyde in TKM buffer (10 mM triethanolamine-HCl, 100 mM KCl and 1 mM MgCl_2_), permeabilized and disrupted by pipetting in 0.25% NP40 in TKM. Individual chromosomes were isolated and fixed with 4% parafomaldehyde in TKM. The chromosomes were then blocked in 2% bovine serum albumin (BSA) in TKM and incubated with antibodies following standard procedures. The secondary antibodies were conjugated to FITC or Texas Red. The immunostained chromosomes were mounted in Vectashield (Vector Laborarories).

### Acquisition and Processing of Confocal Images

Preparations were analyzed and images were taken with a laser scanning microscope (model LSM 510; Carl Zeiss MicroImaging, Inc.) equiped with PlanApochromat objectives 40×/1.0 oil and 63×/1.4 oil, using immersion oil Immersol 518F (Carl Zeiss MicroImaging, Inc.). The optical sections were approximately 1 µm thick. Photoshop software (Adobe) was used for the preparation of composite images and for adjustment of intensity and contrast.

### Immuno-Electron microscopy

Salivary glands were prefixed and permeabilized, and the polytene chromosomes were isolated by pipetting in the same way as those intended to be used in immunofluorescence experiments. The isolated chromosomes were fixed with freshly prepared 4% paraformaldehyde in TKM. The chromosome preparations were blocked in 2% BSA in TKM for 30 min, incubated with primary antibody, washed and incubated with an anti-rabbit IgG conjugated to 6-nm gold particles. The control chromosomes were incubated with either secondary antibody only or with a pre-immune serum. The stained chromosomes were fixed with 2% glutaraldehyde and embedded in Agar 100. Thin sections (70 nm) of plastic-embedded chromosomes were stained with uranyl acetate and examined in a FEI 120 kV TECNAI electron microscope. Images were recorded using a Gatan US 1000P CCD camera. For quantitative purposes, the BR genes were photographed at random areas and the numbers of gold markers in the proximal, middle, and distal segments of the BR genes were counted. The specificity of the immuno-EM results was supported by negative control preparations that were processed in parallel and incubated with either the pre-immune serum or with only secondary antibody. The labeling density in the negative controls was calculated and was found to be between 18.5 and 7.5%, respectively.

### Preparation of Nuclear Protein Extracts

*C. tentans* tissue culture cells, *Drosophila* S2 cells or human HeLa cells were homogenized in PBS containing 0.2% NP-40. The homogenate was centrifuged at 1500 *g* for 10 min at 4°C. The pellet containing the nuclei was resuspended in PBS, sonicated and centrifuged at 16,300 *g* for 10 min at 4°C. The resulting supernatant was the soluble nuclear extract. The pellet was resupended in PBS, digested with RNase A (100 µg/ml) and centrifuged at 16,300 *g* for 10 min at 4°C. The supernatant was the chromosomal RNP fraction and contained proteins that were retained in the pellet through RNA-dependent interactions. For the experiment shown in Figure 4G, the RNase A digestion was allowed to run for 15 min at either 4°C or 25°C.

### Immunoprecipitation

Immunoprecipitation experiments were carried out following standard procedures. Soluble nuclear extracts and chromosomal RNP extracts were prepared as described above, supplemented with 0.1% NP40 and used as input. The bound proteins were eluted, precipitated with acetone and analyzed by SDS-PAGE and Western blotting.

### SDS-PAGE and Western Blotting

Protein extracts were separated by SDS-PAGE and transferred to polyvinylidenefluoride membranes (Millipore) following standard procedures. The NBT/BCIP system was used for detecting secondary antibodies conjugated to alkaline phosphatase. The ECL system (GE Healthcare) was used for the chemiluminiscent detection of horseradish peroxidase.

### Chromatin Immunoprecipitation

Chromatin was prepared from S2 cells after cross-linking with 2% formaldehyde. The chromatin was sheared by sonication to a DNA size of 250–1000 bp and pre-cleared. Chromatin fragments were precipitated with antibodies against either rBrg1 (Ab1) or Pol-II (Abcam) using protein A/G-Sepharose beads (50% of each). The precipitated DNA fragments were purified and amplified by PCR using primers for the CG8092, CG8421 and CG9380 genes. Actin 5C (CG4027) was used as a control. The PCR conditions were optimized to avoid saturation. See

### Microarray Data Analysis

The microarray data was extracted from Array Express, E-TABM 169 (http://www.ebi.ac.uk/microarray-as/aew/). *Drosophila* Genome 2.0 Arrays (Affymetrix) were hybridized with total RNA purified from *Drosophila* S2 cells treated with dsRNA corresponding to dBrm or to other subunits of SWI/SNF [28].

### RNA Interference in S2 Cells

dsRNAs against dBrm and GFP were prepared by *in vitro* transcription from PCR products with T7 promoters on both ends of the amplimers, using the Megascript RNAi kit (Ambion). The sequences of the PCR primers are provided in the Supplementary Materials and Methods. The RNAi treatment was performed as described by Clemens et al. [57]. In brief, 20 µg of dsRNA was applied to S2 cells and the cells were harvested after 48 h. Total RNA from S2 cells was extracted, reverse transcribed and used as a template for PCR reactions using primers specific for selected transcripts. Quantitative real-time PCR was carried out in an ABI7000 system using SYBR Green (Applied Biosystems).The RNAi experiments were repeated three times to confirm the reproducibility of the observations.

### Supporting Information

A detailed description of the Materials and Methods, including primer sequences, are provided as Supporting Information (Text S1). Seven Supporting Figures are also provided (Figures S1, S2, S3, S4, S5, S6, and S7).

## Supporting Information

Figure S1 The amino acid sequence of ctBrm. Multiple sequence alignment of the carboxy terminal portion of the Brm proteins of *C. tentans* (ctBrm, FM211186), *D. melanogaster* (dBrm, CG5942), *Anopheles gambiae* (agBrm, XM_311484) and *Aedes aegypti* (aaBrm, XM_001650039). The multiple sequence alignment was done with using CLUSTAL W at the Biology WorkBench 3.2 (http://workbench.sdsc.edu/). The ctBrm sequence was deduced from a partial cDNA obtained by nested PCR using degenerate primers based on the amino acid sequences of dBrm and agBrm as described in the Supporting Materials and Methods. The amino acid sequence of the carboxy terminal portion of ctBrm shares 60.4% identity with dBrm and 69% with aaBrm and agBrm.
(1.14 MB JPG)

Figure S2 Specificity of three anti-Brm/Brg1 antibodies in *C. tentans*. (A) Three independent antibodies were tested by Western blot against nuclear protein extracts prepared from *C. tentans* cultured cells. Ab1 (*lane 2*) was raised against the rat Brg1 protein. Ab2 (*lane 3*) was raised against the C-terminal part of the ct-BRM protein (Figure S1). Ab3 (*lane 4*) was raised against dBrm. A negative control without primary antibody was processed in parallel (*lane 1*). The three antibodies detected a major band of approximate molecular mass 200 kDa (*arrow*). The mobility of molecular mass standards is shown to the left in kDa. (B) A preparation of total proteins from larval salivary glands was probed with Ab1. The antibody recognized a band with the expected mobility of ctBrm (*arrow*). (C) A nuclear protein extract was prepared from *C. tentans* cultured cells and ctBrm was immunoprecipitated using the Ab1 antibody. A negative control immunoprecipitation without primary antibody was processed in parallel to assess the specificity of the experiment. The immunoprecipitated protein was probed by Western blot using Ab2 and Ab3, as indicated in the figure. Ab2 and Ab3 detect the 200 kDa protein immunoprecipitated by Ab1.
(0.14 MB JPG)

Figure S3 The association of ctBrm with the polytene chromosomes studied by immunofluorescence. (A–B) The images show confocal sections of isolated polytene chromosomes stained with either Ab2 or Ab3, as indicated. Multiple loci were intensely stained in the chromosomes, including the BR puffs BR1, BR2 and BR3 in chromosome IV. (C–D) Confocal sections of isolated polytene chromosomes stained with either Ab2 or Ab3 and co-stained with a mAb against Hrp45. Hrp45 is an hnRNP protein used as a marker to visualize the BRs. The merged images show that the BR puffs are stained by the anti-Brm antibodies. The scale bars represent approximately 10 µm.
(0.33 MB JPG)

Figure S4 Patterns of alternative pre-mRNA processing affected by dBrm depletion. The figure summarizes the different types of alternative pre-mRNA processing reactions changed in S2 cells treated with Brm-dsRNA: (A) intron retention and alternative use of polyadenylation signals, (B) alternative 3′ slice sites and alternative polyadenylation, (C) alternative use of polyadenylation signals, (D) alternative 5′ slice sites and alternative polyadenylation, and (E) exon skipping. Note that the alternative splicing and polyadenlation events are exclusive in most cases and the choice of a given splice site determines the site of a given polyadenylation and vice versa. Examples of representative genes are given for each case to the right. The figure is based on the annotations available at FlyBase (http://flybase.bio.indiana.edu/).
(0.18 MB JPG)

Figure S5 The abundance of alternative CG8421 transcripts is affected by Brm depletion. (A) Schematic representation of the alternative transcripts derived from the CG8421 gene. F and R indicate the positions of the forward and reverse PCR primers, respectively. (B) dBrm was knocked-down in S2 cells by RNAi. Control cells were treated in parallel with dsRNA for GFP, as in Figure 5. RT-PCR reactions were carried out using primers F and R to amplify simultaneously the alternatively spliced CG8421 mRNAs. The PCR products were analyzed in agarose gels stained with ethidium bromide. The mobility of molecular mass standards is shown to the left, in nt.
(0.25 MB JPG)

Figure S6 Optimization of PCR reactions for the proximal, middle and 3′ end regions of the CG9380, CG8092 and CG8421 genes. DNA purified from ChIP experiments was analyzed by PCR using primer pairs specific for each region of interest. For each primer pair, different amounts of DNA template were tested as indicated in the figure, and the conditions of the PCR reactions were optimized in order to determine the linear range of the PCR amplification and to avoid saturation. For each ChIP experiment, the optimal conditions were established and all the samples, including the negative control immunoprecipitation, were run under the same conditions.
(0.45 MB JPG)

Figure S7 The effect of SNR1 depletion on the abundance of alternative transcripts from the CG8421 and CG9380 genes. The expression of SNR1 in S2 cells was silenced by RNAi. Control cells were treated in parallel with GFP-dsRNA. Total RNA was purified from cells treated with either SNR1-dsRNA or GFP-dsRNA, reverse transcribed and analyzed by qPCR. The relative abundance of each mRNA was expressed relative to the actin 5C mRNA levels. (A) The expression of SNR1 was significantly reduced in cells treated by SNR1-dsRNA. (B) Depletion of SNR1 affected the levels of the CG8421 and CG9380 mRNAs. The effects were transcript-specific and were very similar to those observed in BRM-depleted cells (compare with Figure 5C).
(0.20 MB JPG)

Text S1 Supporting materials and methods.
(0.07 MB DOC)
