# Supplementary figures and images for: SWI/SNF Associates with Nascent Pre-mRNPs and Regulates Alternative Pre-mRNA Processing
Source: PLoS Genet. 2009 May 8;5(5):e1000470. doi: 10.1371/journal.pgen.1000470 (PMC2669885; doi:10.1371/journal.pgen.1000470)

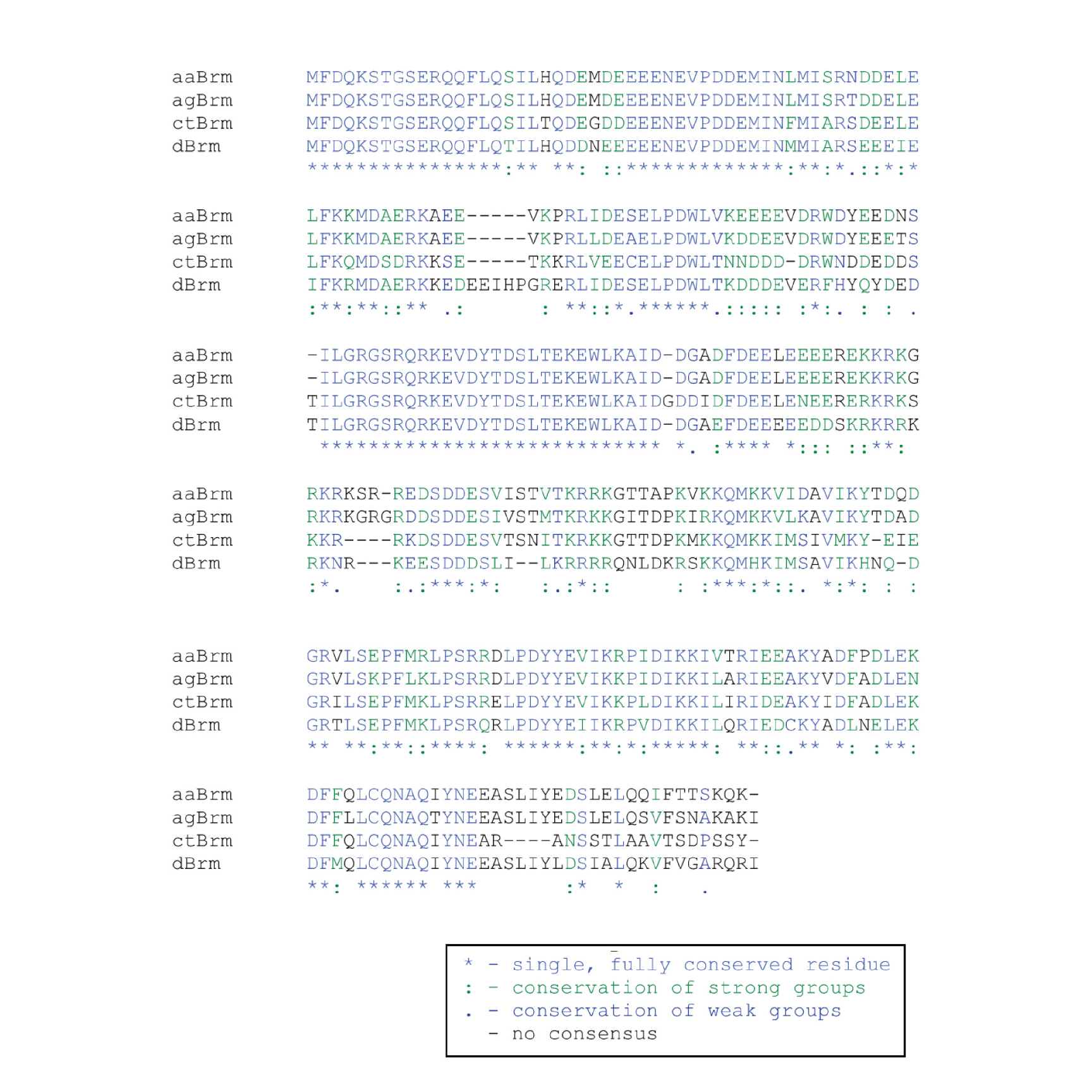

Supplement: Figure S1 — The amino acid sequence of ctBrm. Multiple sequence alignment of the carboxy terminal portion of the Brm proteins of C. tentans (ctBrm, FM211186), D. melanogaster (dBrm, CG5942), Anopheles gambiae (agBrm, XM_311484) and Aedes aegypti (aaBrm, XM_001650039). The multiple sequence alignment was done with using CLUSTAL W at the Biology WorkBench 3.2 (http://workbench.sdsc.edu/). The ctBrm sequence was deduced from a partial cDNA obtained by nested PCR using degenerate primers based on the amino acid sequences of dBrm and agBrm as described in the Supporting Materials and Methods. The amino acid sequence of the carboxy terminal portion of ctBrm shares 60.4% identity with dBrm and 69% with aaBrm and agBrm. (1.14 MB JPG) [file pgen.1000470.s001.jpg]

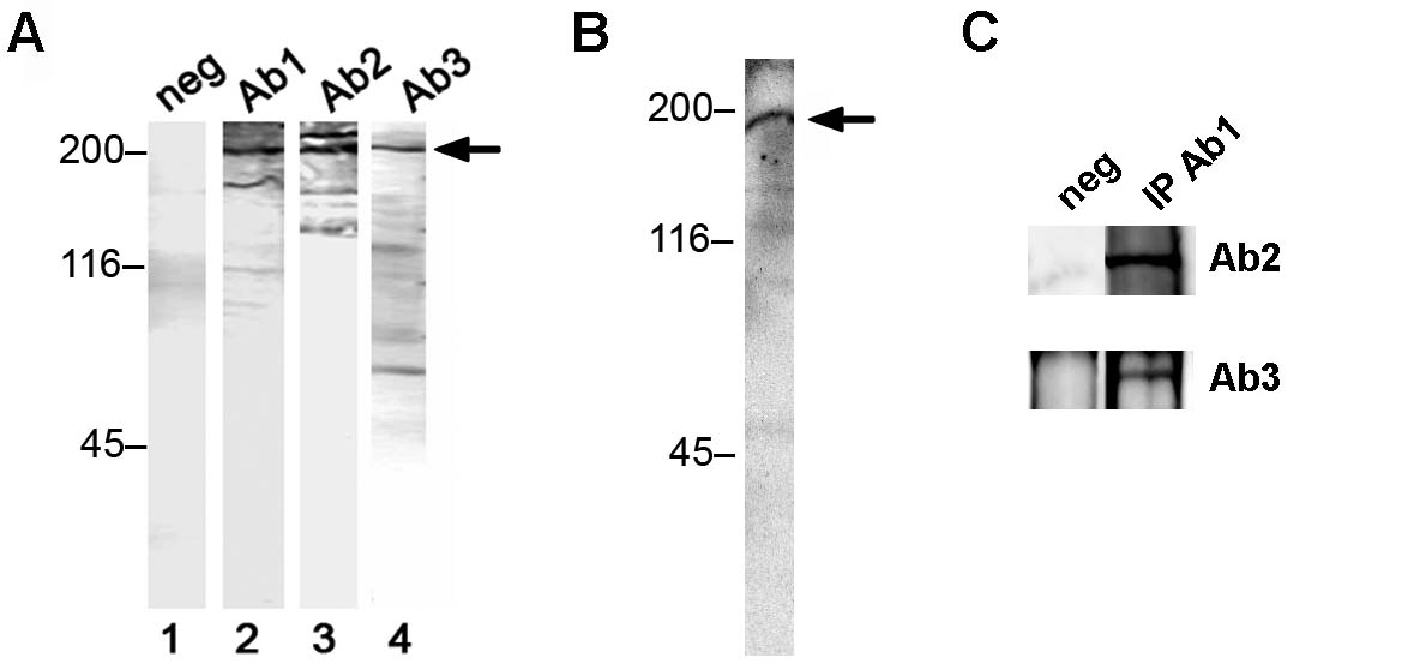

Supplement: Figure S2 — Specificity of three anti-Brm/Brg1 antibodies in C. tentans. (A) Three independent antibodies were tested by Western blot against nuclear protein extracts prepared from C. tentans cultured cells. Ab1 (lane 2) was raised against the rat Brg1 protein. Ab2 (lane 3) was raised against the C-terminal part of the ct-BRM protein (Figure S1). Ab3 (lane 4) was raised against dBrm. A negative control without primary antibody was processed in parallel (lane 1). The three antibodies detected a major band of approximate molecular mass 200 kDa (arrow). The mobility of molecular mass standards is shown to the left in kDa. (B) A preparation of total proteins from larval salivary glands was probed with Ab1. The antibody recognized a band with the expected mobility of ctBrm (arrow). (C) A nuclear protein extract was prepared from C. tentans cultured cells and ctBrm was immunoprecipitated using the Ab1 antibody. A negative control immunoprecipitation without primary antibody was processed in parallel to assess the specificity of the experiment. The immunoprecipitated protein was probed by Western blot using Ab2 and Ab3, as indicated in the figure. Ab2 and Ab3 detect the 200 kDa protein immunoprecipitated by Ab1. (0.14 MB JPG) [file pgen.1000470.s002.jpg]

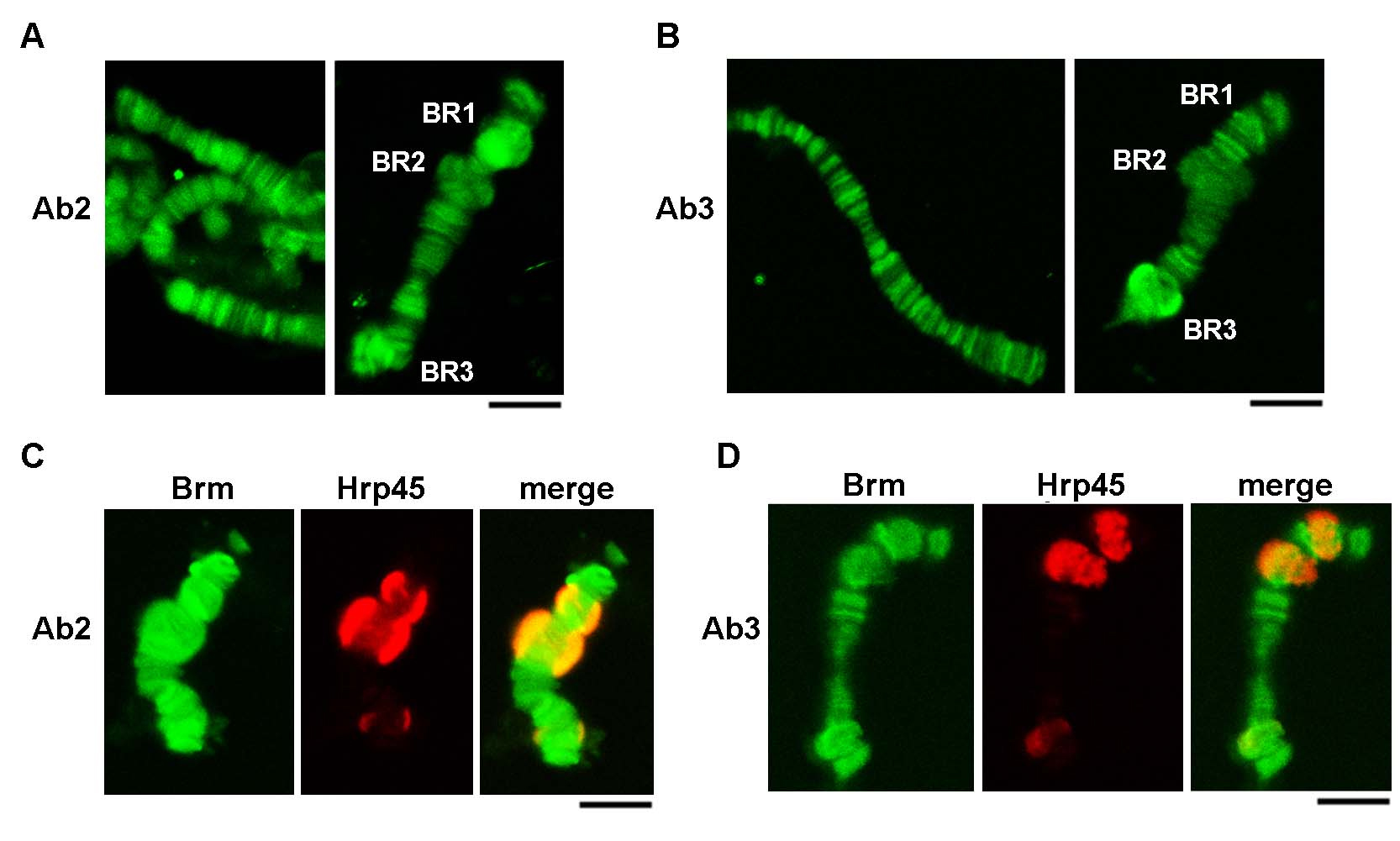

Supplement: Figure S3 — The association of ctBrm with the polytene chromosomes studied by immunofluorescence. (A–B) The images show confocal sections of isolated polytene chromosomes stained with either Ab2 or Ab3, as indicated. Multiple loci were intensely stained in the chromosomes, including the BR puffs BR1, BR2 and BR3 in chromosome IV. (C–D) Confocal sections of isolated polytene chromosomes stained with either Ab2 or Ab3 and co-stained with a mAb against Hrp45. Hrp45 is an hnRNP protein used as a marker to visualize the BRs. The merged images show that the BR puffs are stained by the anti-Brm antibodies. The scale bars represent approximately 10 µm. (0.33 MB JPG) [file pgen.1000470.s003.jpg]

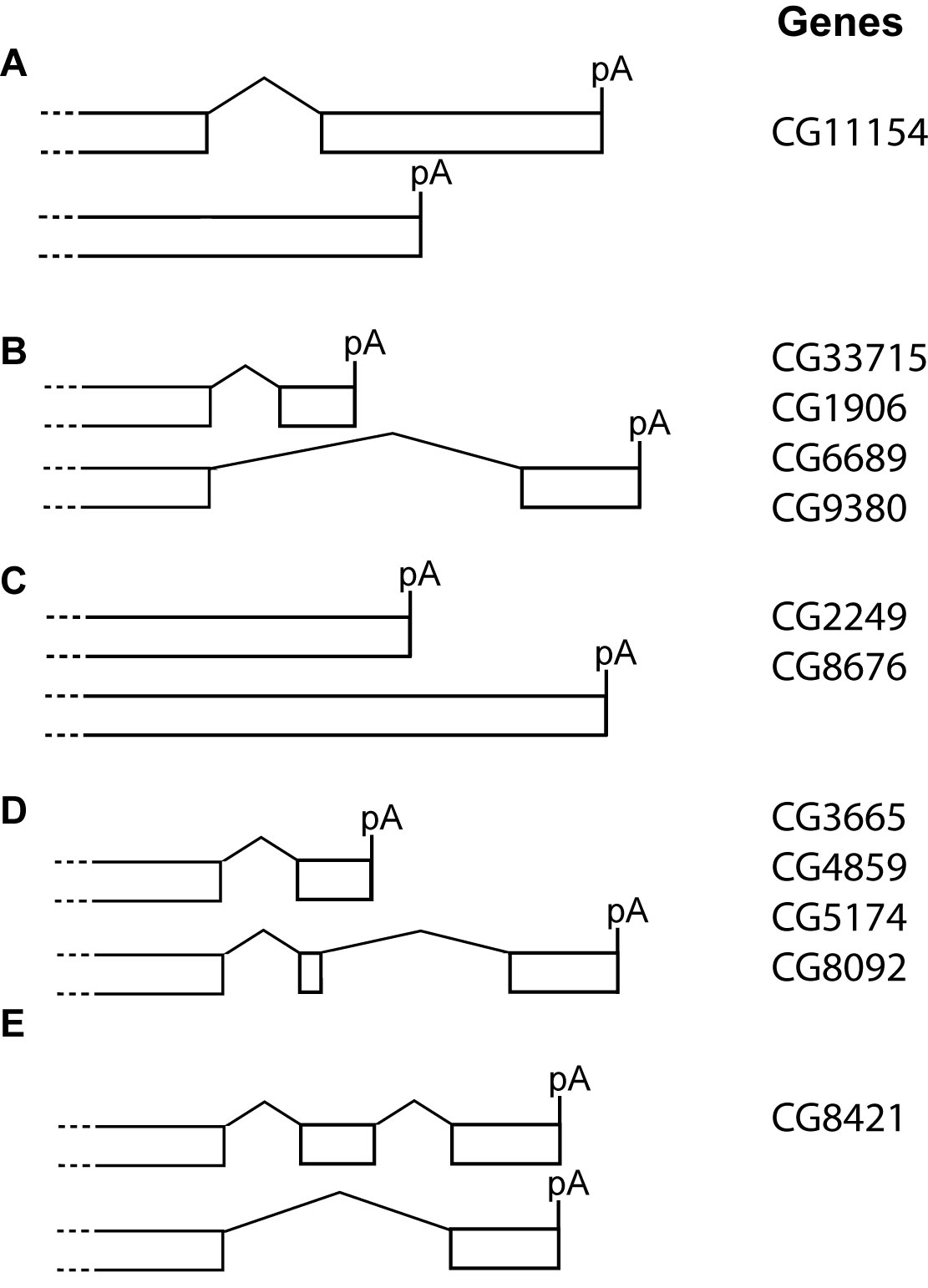

Supplement: Figure S4 — Patterns of alternative pre-mRNA processing affected by dBrm depletion. The figure summarizes the different types of alternative pre-mRNA processing reactions changed in S2 cells treated with Brm-dsRNA: (A) intron retention and alternative use of polyadenylation signals, (B) alternative 3′ slice sites and alternative polyadenylation, (C) alternative use of polyadenylation signals, (D) alternative 5′ slice sites and alternative polyadenylation, and (E) exon skipping. Note that the alternative splicing and polyadenlation events are exclusive in most cases and the choice of a given splice site determines the site of a given polyadenylation and vice versa. Examples of representative genes are given for each case to the right. The figure is based on the annotations available at FlyBase (http://flybase.bio.indiana.edu/). (0.18 MB JPG) [file pgen.1000470.s004.jpg]

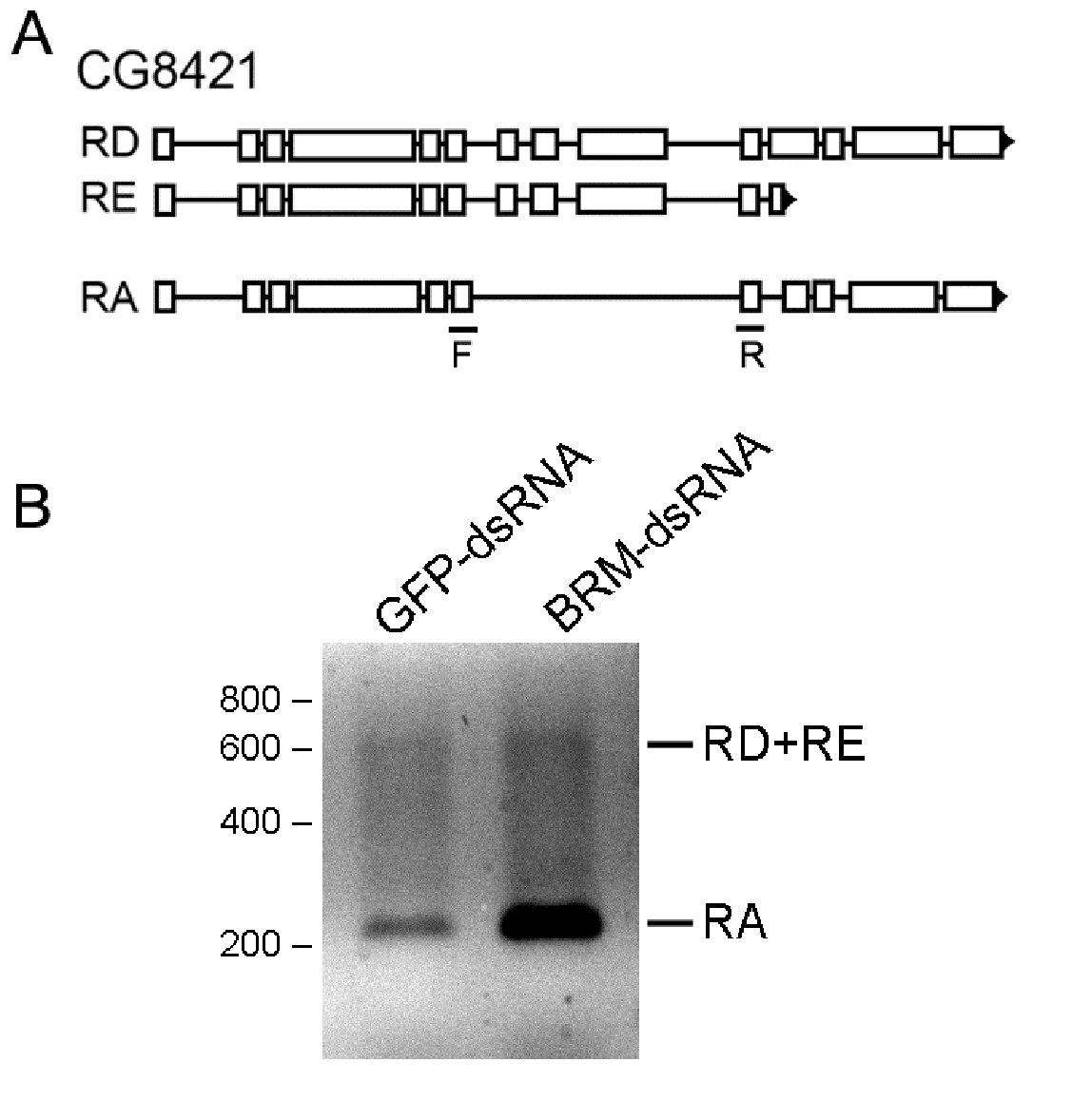

Supplement: Figure S5 — The abundance of alternative CG8421 transcripts is affected by Brm depletion. (A) Schematic representation of the alternative transcripts derived from the CG8421 gene. F and R indicate the positions of the forward and reverse PCR primers, respectively. (B) dBrm was knocked-down in S2 cells by RNAi. Control cells were treated in parallel with dsRNA for GFP, as in Figure 5. RT-PCR reactions were carried out using primers F and R to amplify simultaneously the alternatively spliced CG8421 mRNAs. The PCR products were analyzed in agarose gels stained with ethidium bromide. The mobility of molecular mass standards is shown to the left, in nt. (0.25 MB JPG) [file pgen.1000470.s005.jpg]

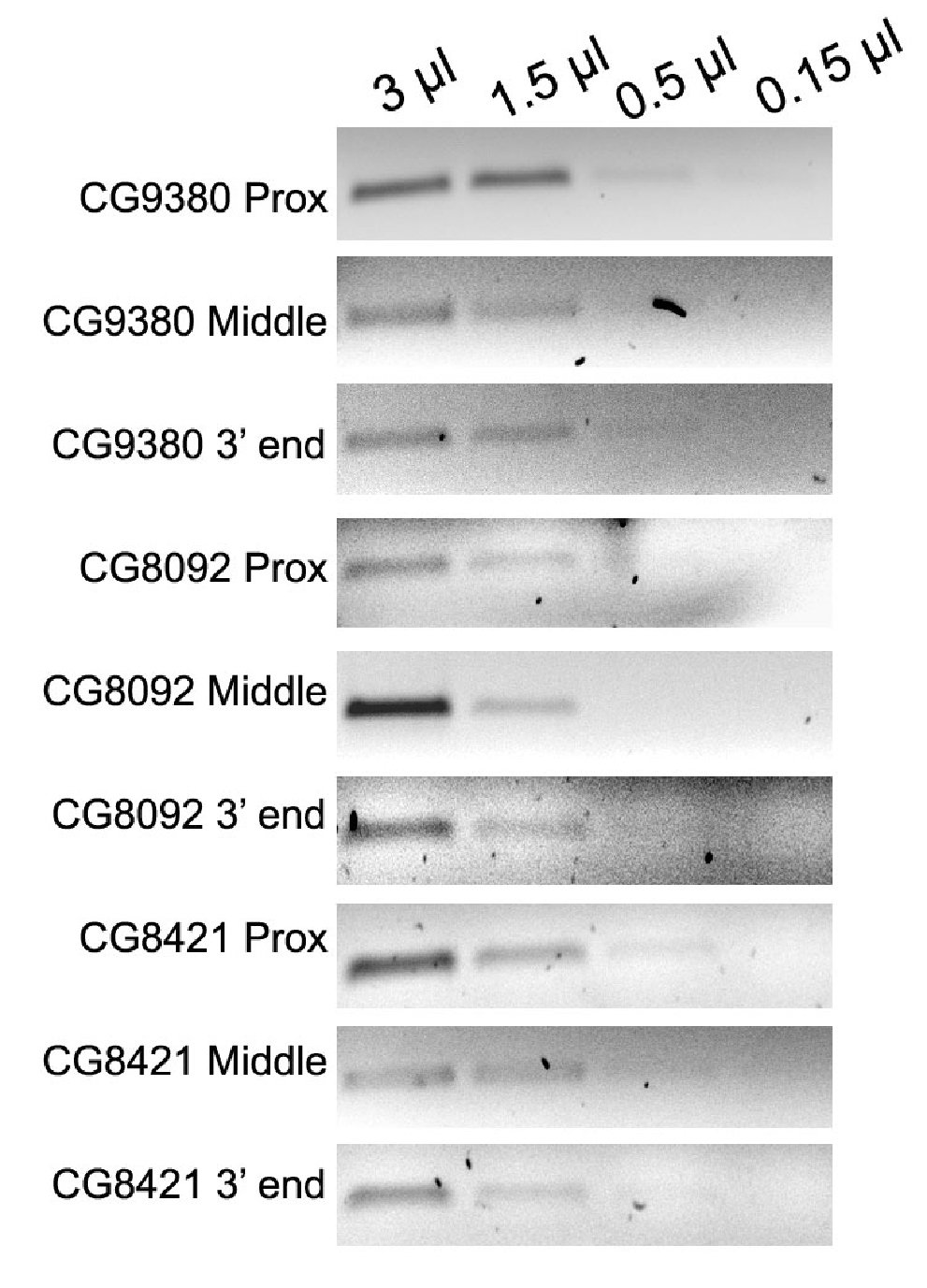

Supplement: Figure S6 — Optimization of PCR reactions for the proximal, middle and 3′ end regions of the CG9380, CG8092 and CG8421 genes. DNA purified from ChIP experiments was analyzed by PCR using primer pairs specific for each region of interest. For each primer pair, different amounts of DNA template were tested as indicated in the figure, and the conditions of the PCR reactions were optimized in order to determine the linear range of the PCR amplification and to avoid saturation. For each ChIP experiment, the optimal conditions were established and all the samples, including the negative control immunoprecipitation, were run under the same conditions. (0.45 MB JPG) [file pgen.1000470.s006.jpg]

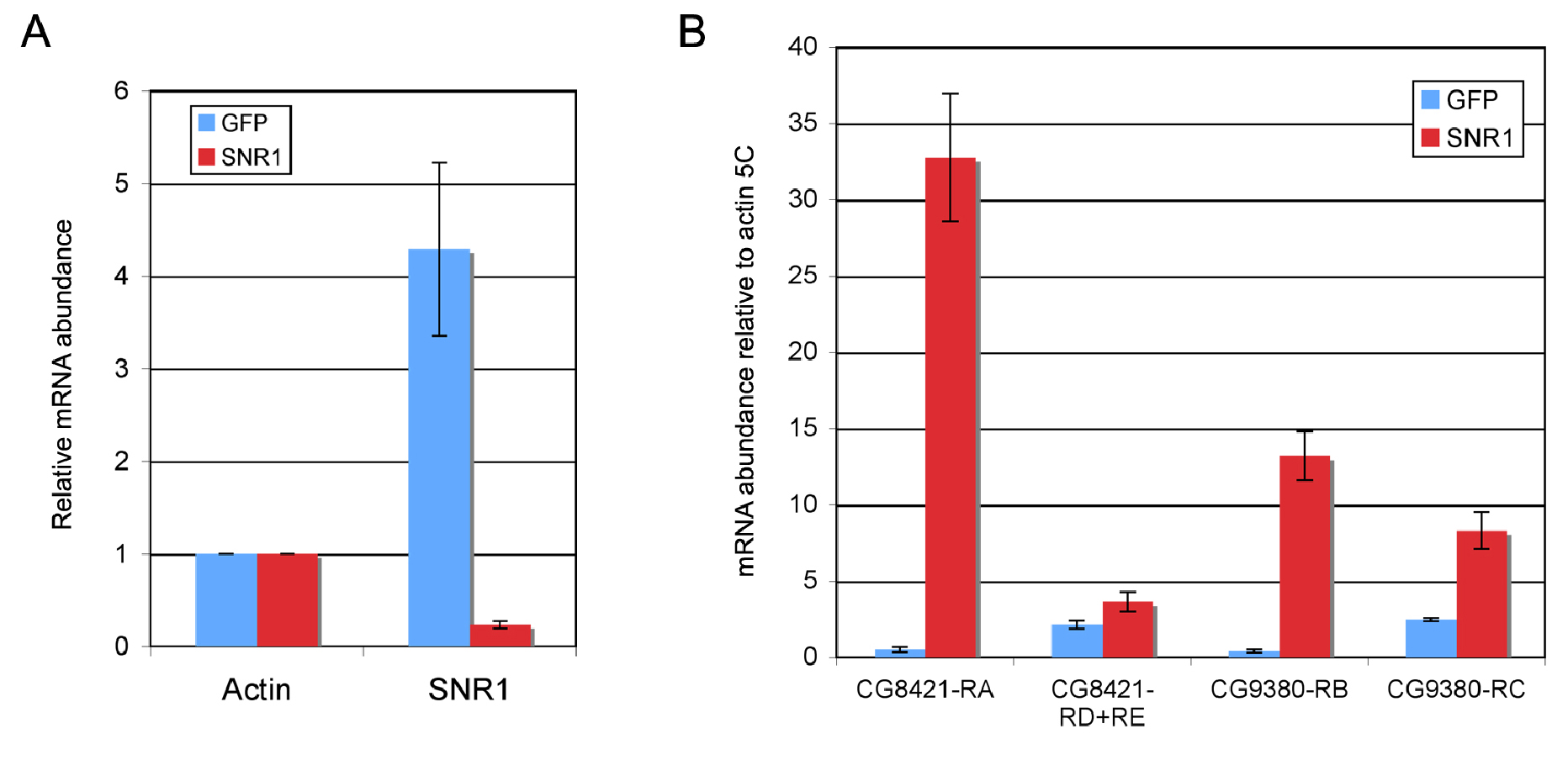

Supplement: Figure S7 — The effect of SNR1 depletion on the abundance of alternative transcripts from the CG8421 and CG9380 genes. The expression of SNR1 in S2 cells was silenced by RNAi. Control cells were treated in parallel with GFP-dsRNA. Total RNA was purified from cells treated with either SNR1-dsRNA or GFP-dsRNA, reverse transcribed and analyzed by qPCR. The relative abundance of each mRNA was expressed relative to the actin 5C mRNA levels. (A) The expression of SNR1 was significantly reduced in cells treated by SNR1-dsRNA. (B) Depletion of SNR1 affected the levels of the CG8421 and CG9380 mRNAs. The effects were transcript-specific and were very similar to those observed in BRM-depleted cells (compare with Figure 5C). (0.20 MB JPG) [file pgen.1000470.s007.jpg]
