# Supplementary material for: SWI/SNF Associates with Nascent Pre-mRNPs and Regulates Alternative Pre-mRNA Processing
Source: PLoS Genet. 2009 May 8;5(5):e1000470. doi: 10.1371/journal.pgen.1000470 (PMC2669885; doi:10.1371/journal.pgen.1000470)
Supplement: Text S1 — Supporting materials and methods. (0.07 MB DOC) [file pgen.1000470.s008.doc]

# Supporting Materials and Methods

**Animals and cell culture**

*Chironomus tentans* were cultured as described by Meyer et al. [1]. The salivary glands used for study were isolated from 4^th^ instar larvae. *C. tentans* tissue culture cells were grown in ZO medium at 24 °C as described by Wyss et al. [2]. *D. melanogaster* S2 cells were cultured at 28 °C in Schneider’s *Drosophila* medium (Invitrogen) containing 10% heat-inactivated fetal calf serum, 50 μg/ml penicillin and 50 µg/ml streptomycin.

**Isolation and expression of a cDNA that encodes ctBrm**

Degenerate primers for nested PCR were designed to match the conserved sequence of the C-terminal part of *D. melanogaster* Brm (CG5942) and *Anopheles gambiae* Brm (AGAP010462). The sequences of the forward primers F1 and F2 were 5´-TAYAARYTNAAYATGGAYGARAA-3´ and 5´-ATHCARGCNGGNATGTTYGAYCARAA-3´, respectively. The reverse primers R1 and R2 were 5´-GCYTCRTTRTANRKYTGNGCRTT-3´and 5´-TTNAYYTCRTARTARTCNGG-3´. The primers were used to amplify Brm-homologous sequences from a total cDNA preparation made from *C. tentans* tissue culture cells using *Taq* Polymerase (Fermentas). A PCR product of about 750 bp was obtained. The PCR product was purified using NucleoSpin extract II columns (Machery-Nagel), ligated into a TOPO vector (Invitrogen) and transformed into Top10 *E. coli* cells. The recombinant plasmid Topo-ctBrm was purified and sequenced at Eurofins MWG Operon (Ebersberg, Germany). The sequence was analyzed using software available at the Biology Workbench (<http://workbench.sdsc.edu/>). The cloned cDNA encoded a partial protein corresponding to amino acids 1252-1455 in dBrm. The insert of the Topo-ctBrm plasmid was further amplified with forward 5´-CCGAATTCATCAACTGGTAGTGAACGTCAA-3´and reverse 5´-GTCCCCAAGCTTTAATTCCCGACGTGATGGCAA-3´ primers containing restriction sites for HindIII and EcoRI, respectively. The PCR product was cleaved, purified and ligated into pET21b (Novagen). The resulting plasmid, pET21-ctBrm, was transformed into BL21 *E. coli* cells (Novagen) for the expression of recombinant protein. The expression was induced with 1 mM IPTG for 2 h at 37 °C. The expressed protein was separated by SDS-PAGE in 15% polyacrylamide gels and stained with Coomassie Brilliant Blue. The band corresponding to recombinant ct-Brm was excised from preparative gels, destained, and used for antibody production in rabbits.

**Antibodies**

The anti-rat Brg1 antibody was raised and characterized by Östlund Farrants et al. [3]. The antibody against the C-terminal part of *ct*-BRM was raised in rabbit at AgriSera (Vännäs, Sweden) according to standard procedures. The anti-dBrm antibody was raised and characterized by Zraly et al. [4]. The monoclonal antibodies 10:3G1 and 2E4 against Hrp36 and Hrp45, respectively, have been previously characterized [5, 6]. The Y12 antibody against the Sm epitope of snRNP core proteins has been characterized by Lerner et al. [7]. The rabbit anti-Hrp59 was the Y38 antibody raised and characterized by Falk et al. [8]. The anti-Pol-II antibody was purchased from Abcam (Ab5408). The anti-TBP was from Santa-Cruz Biotechnology (sc-204). FITC‑conjugated, Texas Red‑conjugated and gold‑conjugated secondary antibodies were from Jackson ImmunoResearch Laboratories. The secondary antibodies conjugated to alkaline phosphatase and horseradish peroxidase were from DakoCytomation.

**Immunofluorescence**

Salivary glands were isolated from 4^th^ instar larve and pre-fixed with 2% formaldehyde in TKM buffer (10 mM triethanolamine-HCl, 100 mM KCl and 1 mM MgCl_2_) for 5 min. The glands were subsequently permeabilized with 2% Nonidet P40 (NP40) in TKM and disrupted by pipetting in 0.25% NP40 in TKM. The chromosomes IV were isolated and transferred to 8-well slides. The chromosomes were washed briefly in TKM and fixed with 4% parafomaldehyde in TKM. The chromosomes were then blocked in 2% bovine serum albumin (BSA) in TKM for 30 min and incubated overnight in a solution of primary antibody diluted in 0.5% BSA in TKM. Affinity purified antibodies were used at 2.5 μg/ml. Sera were diluted 1/2000. Control preparations were incubated with either 0.5% BSA in TKM or pre-immune serum diluted 1/2000 in 0.5% BSA in TKM. The chromosomes were washed with TKM containing 0.01% Tween-20 for 25 min with several changes of buffer and incubated with FITC‑conjugated or Texas Red‑conjugated secondary antibodies for 60 min. The immunostained chromosomes were washed as above and mounted in Vectashield (Vector Laborarories).

**Acquisition and processing of confocal images**

Preparations were analyzed and images were taken with a laser scanning microscope (model LSM 510; Carl Zeiss MicroImaging, Inc.) equiped with PlanApochromat objectives 40x/1.0 oil and 63x/1.4 oil, using immersion oil Immersol 518F (Carl Zeiss MicroImaging, Inc.). The optical sections were approximately 1 µm thick. Photoshop software (Adobe) was used for the preparation of composite images and for adjustment of intensity and contrast.

**Immuno-electron microscopy**

Salivary glands were prefixed and permeabilized, and the polytene chromosomes were isolated by pipetting in the same way as those intended to be used in immunofluorescence experiments. The isolated chromosomes were transferred to 8-well slides and fixed with freshly prepared 4% paraformaldehyde in TKM. After fixation, the chromosomes were washed in TKM three times for 5 min each, and blocked in 2% BSA in TKM for 30 min. The blocked chromosomes were incubated with primary antibody for 1 h at room temperature or overnight at 4 °C, washed and incubated with an anti-rabbit IgG conjugated to 6-nm gold particles. The control chromosomes were incubated with either secondary antibody only or with the pre-immune serum. The stained chromosomes were fixed again with 2% glutaraldehyde in TKM for 1 h, dehydrated in ethanol and embedded in Agar 100. Thin sections (70 nm) of plastic‑embedded chromosomes were mounted on nickel grids, stained with 2% uranyl acetate in 50% ethanol, washed with 50% ethanol and air‑dried. The preparations were examined and photographed in an FEI 120 kV TECNAI electron microscope using a Gatan US 1000P CCD camera. For quantitative purposes, the BR genes were photographed at random areas. These areas contained portions from multiple BR transcription units. The number of gold markers in the proximal, middle, and distal segments of the BR genes was counted, and the percentage in each segment was calculated. Results from at least two independent chromosomes were pooled. The average labeling in the negative controls labeled with a pre-immune serum was less than 20%. Photoshop software (Adobe) was used for the preparation of composite images and for adjustment of intensity and contrast.

**Preparation of nuclear protein extracts**

*C. tentans* tissue culture cells, *Drosophila* S2 cells or human HeLa cells were homogenized in PBS (137 mM NaCl, 3 mM KCl, 8 mM Na_2_HPO_4_, 2 mM NaH_2_PO_4_ at pH 7.2) containing 0.2% NP-40 and protease inhibitor tablets (Roche Diagnostics) using a glass homogenizer. The homogenate was centrifuged at 1500 *g* for 10 min at 4 °C. The pellet containing the nuclei was resuspended in PBS containing protease inhibitor, sonicated three times for 4-5 sec each time, and centrifuged at 16,300 *g* for 10 min at 4 °C. The resulting supernatant was the soluble nuclear extract. The pellet was resupended in PBS containing protease inhibitors, digested with RNase A (100 μg/ml) at room temperature for 15 min and centrifuged at 16,300 *g* for 10 min at 4 °C. The supernatant was the chromosomal RNP fraction and contained proteins that were retained in the pellet through RNA-dependent interactions.

**Immunoprecipitation**

Immunoprecipitation experiments were carried out following standard procedures. Soluble nuclear extracts and chromosomal RNP extracts were prepared as described above, supplemented with 0.1% NP40 and used as input. Primary antibodies, either mAb Y12 or anti-rBrg1, were added to the extracts (final concentration approximately 2 µg/ml) and the samples were incubated for 90 min at 4 °C with gentle agitation. 30 μl of protein G-Sepharose slurry was added and the incubation was continued for additional 90 min at 4 ºC. The beads were washed four times with PBS containing 0.1% NP-40, once with PBS and the proteins were eluted with 1% SDS at room temperature. The eluted proteins were precipitated with acetone and subsequently analyzed by SDS-PAGE and Western blotting.

**SDS-PAGE and Western blotting**

### Protein extracts were separated by SDS-PAGE using the Mini-Protean II system (BioRad) and transferred to polyvinylidenefluoride (PVDF) membranes (Millipore) in Tris-glycine buffer with 0.02% SDS and 4 M urea using a semi-dry electrophoretic transfer cell (BioRad). The membranes were blocked with 10% non-fat dry milk in PBS. The antibodies were diluted in 0.05% Tween-20 and 1% milk in PBS and antibody incubations were carried out according to standard procedures. The NBT/BCIP system was used to detect secondary antibodies conjugated to alkaline phosphatase. The ECL system (GE Healthcare) was used for chemiluminiscent detection of horseradish peroxidase.

**Chromatin immunoprecipitation**

ChIP analyses were performed as described by Takahashi et al. [9]. Chromatin was prepared from S2 cells after cross-linking with 2% formaldehyde. The chromatin was sheared by sonication to a DNA size of 250-1000 bp and pre-cleared. Chromatin fragments were precipitated with antibodies against either rBrg1 (Ab1) or Pol-II (Abcam) in 10 mM Tris-HCl at pH 8.0, 1 mM EDTA, 0.5 mM EGTA containing 0.2% DOC and 1% Triton X-100. The bound complexes were pulled-down with protein A/G-Sepharose beads (50% of each). The beads were washed in RIPA buffer (50 mM Hepes at pH 7.6, 1 mM EDTA, 0.7% DOC, 1% NP40, 0.5 M NaCl). Two controls were used: beads only without primary antibody, and rabbit IgG (DakoCytomation). The precipitated DNA fragments were purified and amplified by PCR with specific primers for the CG8092, CG8421 and CG9380 genes. Actin 5C (CG4027) was used as a control. The PCR conditions were optimized to avoid saturation.

**Microarray data analysis**

The microarray data was extracted from Array Express, experiment E-TABM 169 carried out by Moshkin et al. [10] (<http://www.ebi.ac.uk/microarray-as/aew/>). *Drosophila* Genome 2.0 Arrays (Affymetrix) were hybridized with total RNA purified from *Drosophila* S2 cells treated with dsRNA corresponding to dBrm or to other subunits of SWI/SNF. Mock RNAi experiments were carried out in parallel [10]. We selected genes with multiple probe sets (974 genes). Among them, we selected genes that showed changed expression levels specific for a subset of alternatively processed transcripts with p values below 0.02.

**RNA interference in S2 Cells**

Double-stranded RNAs (dsRNAs) against dBrm and GFP were prepared by *in vitro* transcription from PCR products with T7 promoters on both ends of the amplimers, using the Megascript RNAi kit (Ambion). The sequences of the PCR primers are provided below. The RNAi treatment was performed as described by Clemens et al. [11]. In brief, 20 µg of dsRNA was applied to S2 cells and the cells were harvested after 48 h. Total RNA from S2 cells was extracted using the RNAqueous kit (Ambion). Reverse transcription was performed with Superscript-III (Invitrogen) and hexamer primers (Roche) on 5 µg total RNA. The resulting cDNA was used as a template for PCR reactions using primers specific for selected transcripts. Each RNAi experiment was repeated three times to confirm the reproducibility of the observations.

# Quantitative RT-PCR

Quantitative RT-PCR was carried out in an ABI7000 system using SYBR Green (Applied Biosystems). The sequences of the primers used for the analysis are given below. Each transcript was quantified from two independent RNAi experiments, and each experiment was quantified in triplicate. For each transcript, the relative abundance was determined on the basis of the threshold cycle (Ct). The values obtained were expressed relative to actin 5C analyzed in parallel. The results presented in the histograms are averages and the error bars show standard deviations from three PCR determinations.

**Primer sequences**

Primers used for the synthesis of dsRNA

Brm-dsRNA (two different regions targeted)

BKN F: TGAACTGTATCAGCCGCTTG

BKN R: AAGCCCAATCGCATTACAAC

HFA F: GTTTCGCTGTACAATAACAATC

HFA R: ATGTGGAGCAGGACTTAAAG

GFP-dsRNA:

GFP F: ATGGTGAGCCAAGGGCGAGGAGCTG

GFP R: GCGGTCACGAACTCCAGCAG

Primers used for RT-PCR analysis

Actin F: GAACCACTCCCACCAAGAAA

Actin R: TGATCACTTTCAGCACTGGC

18S F: AGCTAGCAATTGGGTGTAGC

18S R: TGAGTCTCGTTCGTTATCGG

Asph1 F1: CCTGGCTCAGCAATTCGAAAA

Asph1 R1: CAACTCTTGCTCGAAAGGAT

Asph1 F2: GATCGAAGAATCAGTAGAGCCG

Asph1 REE2: TCTTTCGGCTTTATTTGTGATGCCAACC

CG9380 F1: GCGAATCGCTATTTGACCAT

CG9380 REE1: TCCATATTTCGCACATACGT

CG9380 F2: CAACTACCAGTGTCGAATGGCAAT

CG9380 REE2: AACGATTGTGGCACATACGT

G8092 F1: TTATGCGGAGAAGTCCATCC

CG8092 R1: AGTGAGAAACGCAGGCACTT

CG8092 REE: TGGTTGCCACCTGATACAGAC

Primers used for quantitative RT-PCR analysis

qActin 2F: GCACACCCACAAGCTTACACA

qActin 2R: TTGCGCTTTGGGAAATATCTTC

qAsph F1: CCTGGCTCAGCAATTCGAAAA

qAsph R1: CAACTCTTGCTCGAAAGGAT

qAsph RDRE F: TATGTGCCCGAGACATTTGA

qAsph RDRE R: ATCGCTGTGACCTACTGGCT

q8092 RA F1: ATTGCCGAGCAGACAAGAGT

q8092 RA R1: CAGTCCTTGAACGGATTGGT

q8092 RB F2: GCCGTGAATAGAATAGTGCG

q8092 RB R2: CTCAGCTGTTGGGTTCGATT

q9380 RC F: TTGGCGAGGTTTCATGTGTA

q9380 RC R: AACCACAGAAGCTAAGGCCA

q9380 RB F2: ACGATCTTTCAAATGCCTGC

q9380 RB R2: ACAGGACGTGGTGTTTGATG

ds SNR1 MRC F: AGGGTCAAAAGCTGCGCGACACCTT

ds SNR1 MRC R: GGTCCTGGTCGCGGATTTTCTTCTC

ds SNR1 MRC F T7: TAATACGACTCACTATAGGGAGAAAGGGTCAAAAGCTGCGCGACACCTT

ds SNR1 MRC R T7: TAATACGACTCACTATAGGGAGAAAGGTCCTGGTCGCGGATTTTCTTCTC

SNR1 Control F3: ACAGGGCATTGAGAGACCAC

SNR1 Control R3: GGTCATTCCCGGGTACTTCT

## Primers used for ChIP

Actin F: GAACCACTCCCACCAAGAAA

Actin R: TGATCACTTTCAGCACTGGC

CG8092 Prox F: ATCCACGCAAGGAAAGCTAA

CG8092 Prox R: ACCCAGAACTCCATGTCCAG

CG8092 Middle F: CAACCAATCCGTTCAAGGAC

CG8092 Middle R: CTCACGTGTTGGGTTCGATT

CG8092 End F: CATATTCATCGCACATTGGC

CG8092 End R: AATCCCAACTCACATCCAGC

CG8421 Prox F: CGCTCGAAAAGGAAAAACAA

CG8421 Prox R: TCACACCAAATAACGGTCCA

CG8421 Middle F: GTCGCAACCAAAAGGTGTCT

CG8421 Middle R: GGGTCTCTTGAAATGAGCGT

CG8421 End F: GCTTTAGAACCTGGG´CGTGAG

CG8421 End R: ACCGGTCAAATGGGTGATAA

CG8421 Inter F: TGGGTGTGGGTGAATGTATG

CG8421 Inter R: CAGATGGGCGTGTAAATGTG

CG9380 Prox F: GGTGTGGTAGGATGCGGTAG

CG9380 Prox R: TGAAACTTTGCAATCGGTGA

CG9380 Middle F: GCGAATCGCTATTTGACCAT

CG9380 Middle R: CAAGTCTGGACACTGGGTGA

CG9380 End F: CAACTACCAGTGTCGAATGGCAAT

CG9380 End R: AACGATTGTGGCACATACGT

**References**

1. Meyer, B., Mähr, R., Eppenberger, H.M., and M. Lezzi. 1983. The activity of Balbiani rings 1 and 2 in salivary glands of Chironomus tentans larvae under different modes of development and after pilocarpine treatment. Dev Biol. 98:265-277.

2. Wyss, C., 1982. Chironomus tentans epithelial cell lines sensitive to ecdysteroids, juvenile hormone, insulin and heat shock. Exp Cell Res. 139 : 309-319.

3. Östlund Farrants, A.K., Blomquist, P., Kwon, H., and O. Wrange. 1997. Glucocorticoid receptor-glucocorticoid response element binding stimulates nucleosome disruption by the SWI/SNF complex. Mol Cell Biol. 17:895-905.

4. Zraly, C.B., Marenda, D.R., Nanchal, R., Cavalli, G., Muchardt, C., and Dingwall, A.K. (2003). SNR1 is an essential subunit in a subset of Drosophila brm complexes, targeting specific functions during development. Dev. Biol. *253*, 291-308.

5. Kiseleva, E., Visa, N., Wurtz, T., and B. Daneholt. 1997. Immunocytochemical evidence for a stepwise assembly of Balbiani ring premessenger ribonucleoprotein particles. Eur J Cell Biol. 74:407-416.

6. Wurtz, T., Kiseleva, E., Nacheva, G., Alzhanova-Ericcson, A., Rosén, A., and B. Daneholt. 1996. Identification of two RNA-binding proteins in Balbiani ring premessenger ribonucleoprotein granules and presence of these proteins in specific subsets of heterogeneous nuclear ribonucleoprotein particles.Mol Cell Biol. 16:1425-1435.

7. Lerner, E.A., Lerner, M.R., Janeway, C.A. Jr, and J.A. Steitz. 1981. Monoclonal antibodies to nucleic acid-containing cellular constituents: probes for molecular biology and autoimmune disease. Proc Natl Acad Sci U S A. 78:2737-2741.

8. Falk, R., C. Agaton, E. Kiesler, S. Jin, L. Wieslander, N. Visa, S. Hober, and S. Stahl. 2003. An improved dual-expression concept, generating high-quality antibodies for proteomics research. Biotechnol. Appl. Biochem. 38:231-239.

9. Takahashi ,Y., Rayman, J.B., Dynlacht, B.D. 2000. Analysis of promoter binding by the E2F and pRB families in vivo: distinct E2F proteins mediate activation and repression. Genes Dev 14: 804-816.

10. Moshkin, Y.M., Mohrmann, L., van Ijcken, W.F., and C.P. Verrijzer. 2007. Functional differentiation of SWI/SNF remodelers in transcription and cell cycle control. Mol Cell Biol. 27:651-661.

11. Clemens, J. C., Worby, C. A., Simonson-Leff, N., Muda, M., Maehama, T., Hemmings, B. A., Dixon, J. E. 2000. Use of double-stranded RNA interference in Drosophila cell lines to dissect signal transduction pathways. Proc. Natl. Acad. Sci. U. S. A. 97: 6499-6503.
